# Simulation study of the Lower Cretaceous geothermal reservoir for aquifer thermal energy storage

**DOI:** 10.1007/s10653-021-01130-7

**Published:** 2021-10-28

**Authors:** Elżbieta Hałaj, Leszek Pająk, Bartosz Papiernik

**Affiliations:** AGH University of Science and Technology, Mickiewicza 30 Ave, 30-059 Krakow, Poland

**Keywords:** ATES, Numerical simulation, Saturation index, Energy recovery ratio, Increasing geothermal potential

## Abstract

The aquifer thermal energy storage (ATES) has gained attention in several countries as an installation for increasing the energy efficiency of geothermal systems and the use of waste heat. The Lower Cretaceous reservoir is known as one of the most prospective for geothermal purposes in Poland. However, in the southern part of the Mogilno–Łódź Trough (Central Poland) is considered to have a lower geothermal potential. The aim of this paper is to study whether the Lower Cretaceous reservoir in this area is suitable for aquifer thermal energy storage. Prior to dynamic simulations in Feflow© software, a regional Petrel© static parametric model which includes a multidisciplinary approach was prepared. A methodology of fitting Petrel’s structural and parametrical model to Feflow requirements is provided within this paper. The performance simulation of 4 systems has been conducted for 30 years. Increasing precipitation potential is expected for aragonite and calcite along with a temperature increase, while silica precipitation carries a much smaller risk. The paper presents potential for ATES systems in the Lower Cretaceous reservoir of the study area with the best doublet location having thermal recovery ratio of 0.47 and 0.34 for 30 and 40 K temperature differential scenario. An imbalance in heat injection/production in the storage system can cause the reservoir to cool faster than in conventional geothermal heating installation. ATES can provide a successful geothermal reservoir boosting in the case of applying a balanced injection of waste heat.

**Supplementary Information:**

The online version contains supplementary material available at 10.1007/s10653-021-01130-7.

## Introduction

The aquifer thermal energy storage (ATES) installations can be helpful in increasing the energy efficiency of geothermal systems and the use of waste heat. In such a system, an aquifer is used as a storage medium and groundwater is used as the heat carrier fluid (Xu et al., 2014). ATES, as one of the thermal energy storage systems, can bridge the inequality between high-demand and high-energy supply times (Lee, 2013; Fleuchaus et al., 2018). Warm or cold water in shallow alluvial aquifers can be stored during off-peak periods and recovered during peak periods in real-time, intraday and interday frequencies (De Schepper et al., 2019). This kind of storage is especially advantageous for the recovery of waste heat, which would normally be lost.

ATES systems can be coupled with various types of facilities such as hospitals, offices, housing, stores, airports, universities, greenhouses and data centres. ATES systems are often connected with heat pump installations (De Schepper et al., 2020; Birhanu et al., 2015; Vanhoudt et al., 2011). ATES may be also developed with cooling towers of conventional thermal plants to conserve energy and maintain the normal running of such facilities (Xiao et al., 2016). ATES systems are also an attractive solution for urban areas to be coupled with district heating systems (Guelpa and Verda, 2019).

Any long-term prognosis related to ATES systems that focus on forecasting changes with its energy parameters has to consider time-dependent changes of temperature distribution. Mathematical description of heat and mass exchange processes is often led by numerical solutions that help to consider complex geometry and time-dependent boundary conditions (Dendys et al., 2015). Several authors present their concepts from different countries (Franco & Vaccaro, 2014; Pola et al., 2015; Ganguly et al., 2017; Winterleitner et al., 2018; Raguenel et al., 2019; Ma et al., 2020, Zhang et al., 2020). A static model, which can correctly represent the geological nature of the reservoir, is necessary in order to achieve a reliable evaluation and assessment through numerical simulations (Wang & Bauer, 2016). And numerical modelling is a beneficial instrument for evaluating natural processes (Torresan et al., 2020, 2021). Within that kind of research, a multidisciplinary approach is required. In recent years, due to the increased use of energy from renewable sources such as wind or solar power, the geological subsurface is investigated as a georeservoir for renewable energy storage (Bauer et al., 2013).

The simulation of ATES with numerical models has successfully been presented by several authors (Rostampoura et al., 2019; Gunguly et al., 2017; Kim et al., 2010). The thermal effects of geological layering were evaluated by Bridger and Allen (2014). Sommer et al. (2014) assessed the thermal storage performance and the heat transport around the wells. The ATES system was also modelled with the integration of district heating and heat pumps by Todorov et al. (2020).

**Fig. 1:**
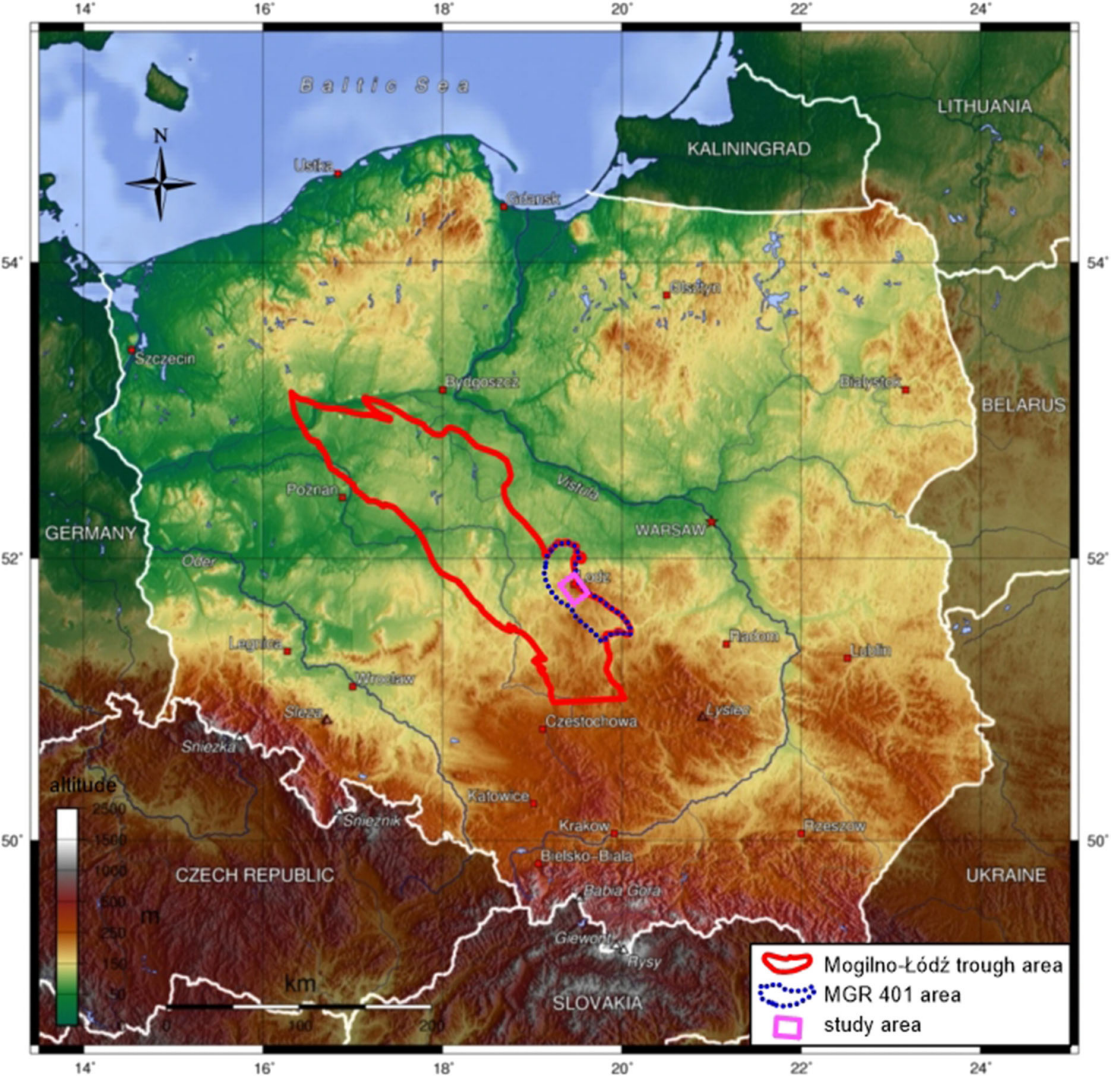
Location of the study area (based on Hałaj (2019) and topographic map of Poland, CC-BY-SA-3.0). MGR 401—Main Groundwater Reservoir No. 401

This research was conducted for the Lower Cretaceous formations in the Mogilno–Łódź Trough. It is located in Central Poland. The study area is considered a part of the trough as a structure in the regional sense. The study area is shown in Fig. 1. The exact location of the research area was chosen in a place where the geological model was available. The study area is located in MGR (Main Groundwater Reservoir) No. 401. MGRs are geological structures or their fragments showing the highest water-bearing capacity and abundance in the hydrological regions, which currently are or may become the main source of water supply for inhabitants. MGRs must meet several quality and quantity requirements (Nowicki, 2007). This may cause several limitations for ATES system locations. However, an ATES system in the area may have a positive impact on water quality monitoring and contamination prevention.

Some geothermal research in the area has been conducted since 1980. The Mogilno–Łódź Trough was mapped in the regional geothermal atlas (Górecki & Hajto et al., 2006). The northern and central area of the trough is considered rich in geothermal waters with a temperature high enough to be useful for heating or bathing purposes. There are several geothermal facilities, including 2 heating plant, 2 bathing and recreation centres and 1 health resort (Halaj, 2015). Researches were also conducted to find the best location for geothermal binary power stations (Bujakowski & Tomaszewska et al., 2014). Some ATES location was pre-considered in eastern ward geological unit—Warsaw Trough (Kępińska et al., 2017c).

The southern part of the trough, being the subject of interest of this research, received less geothermal research attention, partly because of the lower temperature of the water. However, the advantage of this area is also a lower mineralization of the water. The low temperature of water may not be enough for direct geothermal purposes, but it can be used as low-temperature source of energy in case of a heat pump.

This paper evaluates the possibility of ATES in the Lower Cretaceous reservoir located in the southern part of the Mogilno–Łódź Trough. The aim of this work is to study whether the Lower Cretaceous reservoir in the area with lower geothermal potential is suitable for aquifer thermal energy storage by considering some chosen scenarios and their thermal regimes.

The novelty of the study is to simulate ATES systems not only as storage systems but also considering them of increasing the geothermal potential of the area with the use of excess heat.

## Study area

### Geological setting

**Fig. 2:**
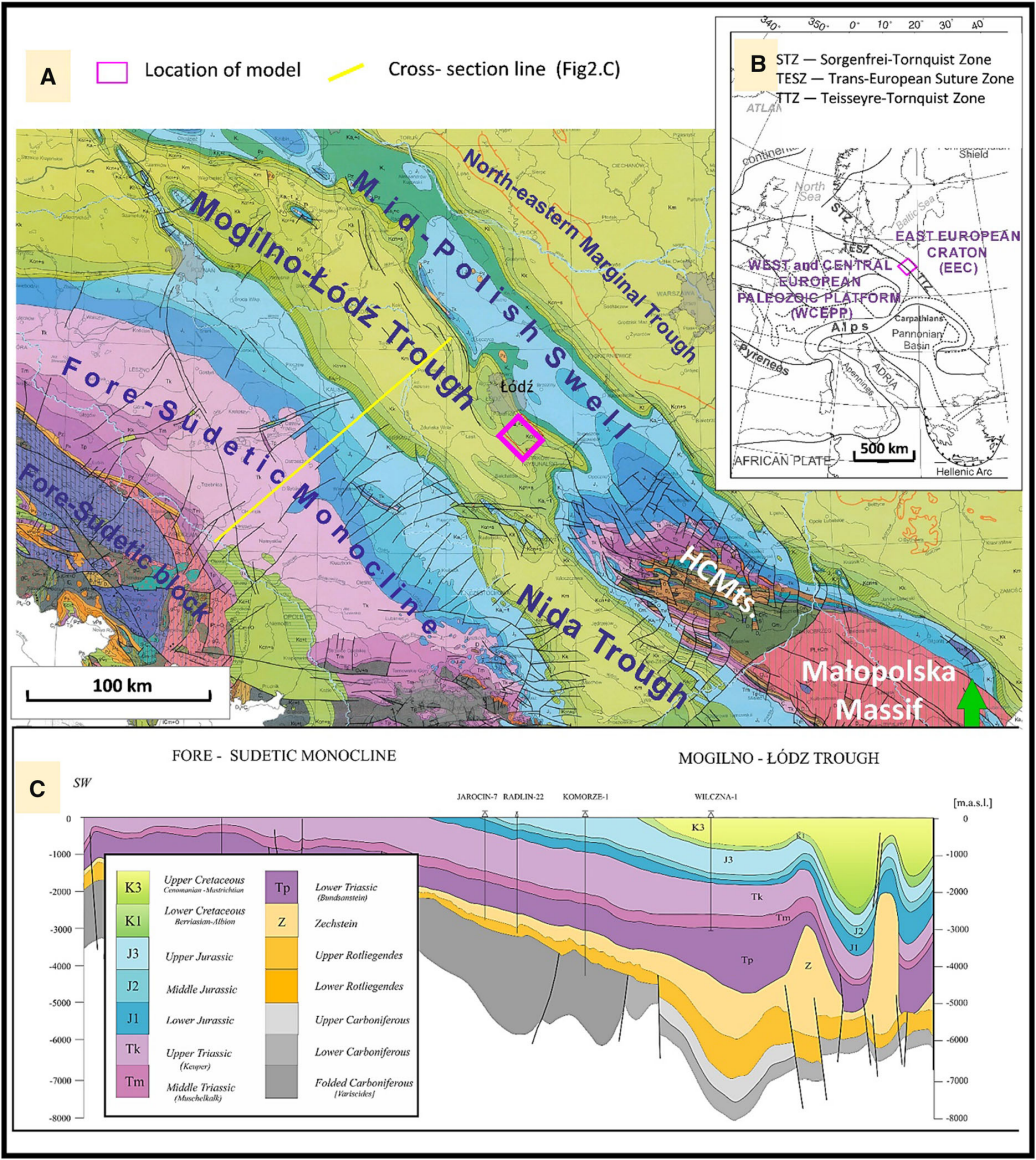
Regional geology of the central part of the Polish Lowlands **a** Cenozoic subcrop map (WMS file based on Dadlez et al. 2000): Colours of Carboniferous to Cretaceous subcrops explained on inset C; **b** tectonic setting of the Polish Lowlands without Cenozoic cover on the background of Europe tectonics (based on Sowiżdżał et al. 2013); **c** geological cross section through the central part of Mogilno–Łódź trough (based on Pletsch et al., 2010)

The research area is located in the marginal part of the West and Central European Palaeozoic Platform, including a set of terranes like Trans-European Suture Zone (TESZ) (Fig. 2) (Królikowski, 2006; Guterch et al., 2010). This zone corresponds to the location of the axial part of the Polish Basin–Mid-Polish Trough (MPT), which was the eastern termination of the Permian–Mesozoic system of epicontinental basins of Western and Central Europe (Ziegler, 1990a; Pharaoh et al., 2006; Krzywiec, 2006; Jarosiński et al., 2009). MPT was created as a result of long-term thermal subsidence, which comprised three major extensional pulses from the Zechstein to Scythian, the Oxfordian to Kimmeridgian and the early Cenomanian (Dadlez et al., 1995; Stephenson et al., 2003). The regional subsidence patterns of MPT was locally superimposed by salt movements, which within the central (Kuiavian) part of the trough, started in the Early Triassic (Krzywiec, 2004a, b; 2006). Zechstein salts created a complex system of salt structures developed in the central and north-western segments of the MPT (Pożaryski, 1977; Krzywiec, 2004a, b). The final stage of the Mid-Polish Trough evolution was Late Turonian–Paleocene compressional inversion (Dadlez et al., 1995; Krzywiec, 2006; Jarosiński et al., 2009; Leszczyński, 2010). The MPT’s inversion was a widespread uplift typical for basins with thick salts. Inversion and subsequent erosion created the present-day tectonic pattern of Polish Lowlands including Fore-Sudetic Monocline, Szczecin Mogilno–Łódź–Nida Trough, Mid-Polish Anticlinorium (Swell) and North-Eastern Marginal Trough (Fig. 2).

**Fig. 3:**
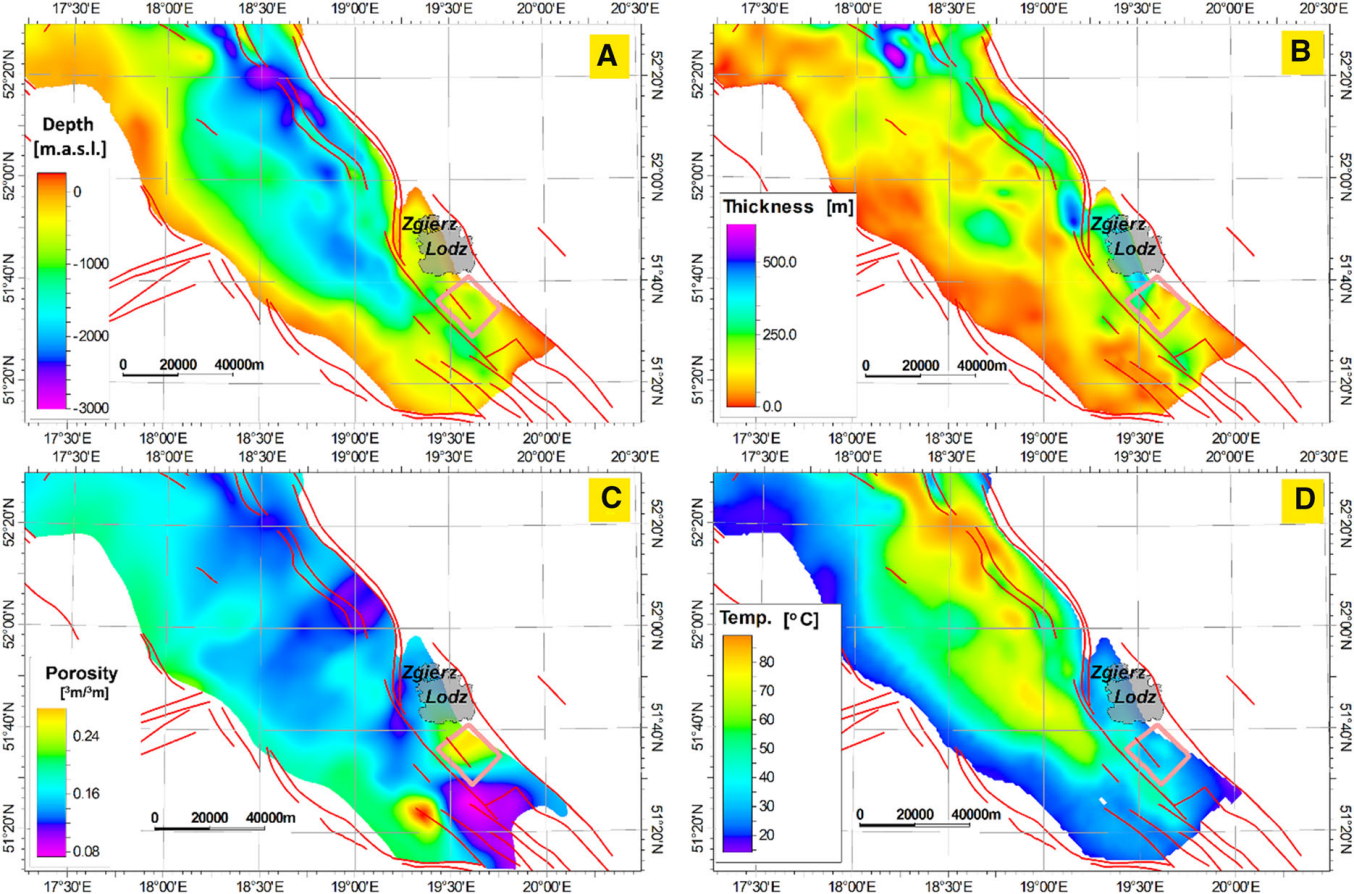
Geological and thermal conditions of the Lower Cretaceous in the Southern Part of the Mogilno Trough and in the Łódź Trough. **a** Structure map of the top surface; **b** variability of thickness **c** average porosity; **d** average temperature (Kępińska et al. 2017a, b, c). Model location marked with a pink box

The presented model is located at the top part of the Tuszyn anticline, which is salt brachyantycline located in the NE part of the Łódź Trough, close to the Mid-Polish Swell and about 10 km SE of Łódź (Górecki and Hajto et al., 2006; Wójcicki, 2012; Kępińska et al., 2017a; b). Two main geothermal complexes were identified in this geological unit—sandstones of Lower Cretaceous and Lower Jurassic. Additionally, in the Tuszyn Anticline highly permeable aquifer was identified in Middle Jurassic sandstones. In the presented paper, Cretaceous reservoir was selected for the further detailed studies. As shown in Fig. 3 in the Mogilno–Łódź Trough, the Lower Cretaceous formations are located at various depths which range from 0 to 3,010 m bsl (below sea level).

Lower Cretaceous profile in the Tuszyn anticline is represented by the sandy deposits of Lower Cretaceous characterized by the Middle Albian–Barremian, Hauterivian and Valanginian. The best reservoir interval comprises the Mogilno Formation (Fm.), (Middle Albian–Barremian) represented by the Pagórczańskie Member (Mb.) (sandstones), the Gopło Mb. (mudstones and sandstones) and the Kruszwica Mb. (sandstones).

The Upper Cretaceous deposits are in general, a thick caprock complex. They represent six individual transgressive–regressive cycles in the Polish Basin (Leszczyński, 2010). K3-II–K3-III (Early–Late Cenomanian) is represented by a siliciclastic and siliciclastic–carbonate deposition to open-marine carbonate shelf deposition. K3-IV (Late Cenomanian–Middle Turonian) is developed as carbonate and carbonate-siliceous lithofacies. The K4-I (Late Turonian–Coniacian) cycle is composed of carbonate-siliceous lithofacies especially various types of opokas. K4-II (Santonian–Early Campanian) contains open-marine carbonate–siliceous lithofacies, similarly as Cycle K4-III (Early Campanian–Early Maastrichtian).

### Hydrogeological setting

According to regional structural and parametric modelling results (Kępińska et al., 2017a; b, c), the Lower Cretaceous reservoir is known as one of the most prospective for geothermal purposes. According to (Kępińska et al., 2017c; Sowiżdżał et al., 2020), this reservoir can have a thickness from 0 to 630 m. At the top part of the Tuszyn anticline, the total thickness of the formation is 81–95 m, while in its eastern limb, it increases up to 120.5 m (Tuszyn 3). Kruszwica Mb. displays a thickness from 61 to 105 m, containing ca. 10-m-thick complex of mudstones and clays. The Gopło Mb. thickness changes from 6 to 22.5 m.

The Lower Cretaceous sandstones in the research area have the average temperature ranges from 21 to 50 °C, porosity from 0.07 to 0.3 (Fig. 3) and average permeability from 0.5 to 1,000 mD, assuring the good quality of the aquifer (Kępińska et al., 2017a). These are of mean values calculated from the 3D model. These are average value calculated for spatially equivalent (XY coordinates) nodes of 3D grid and display in each grid node of the map (2D grid). The input raw data measured on the samples may therefore have a larger range of variability, especially for permeability. In case of regional statistical description, it was intended to show typical values not extremes.

The mineralization of waters from the Mogilno–Łódź Trough differs and can reach high values from a few to 100 g/L. The most common types of waters in the area are Na–Cl and Na–(Ca)–(Cl)–HCO_3_. The potential, maximum discharge of the wells is assessed to 400 m^3^/h.

**Fig. 4:**
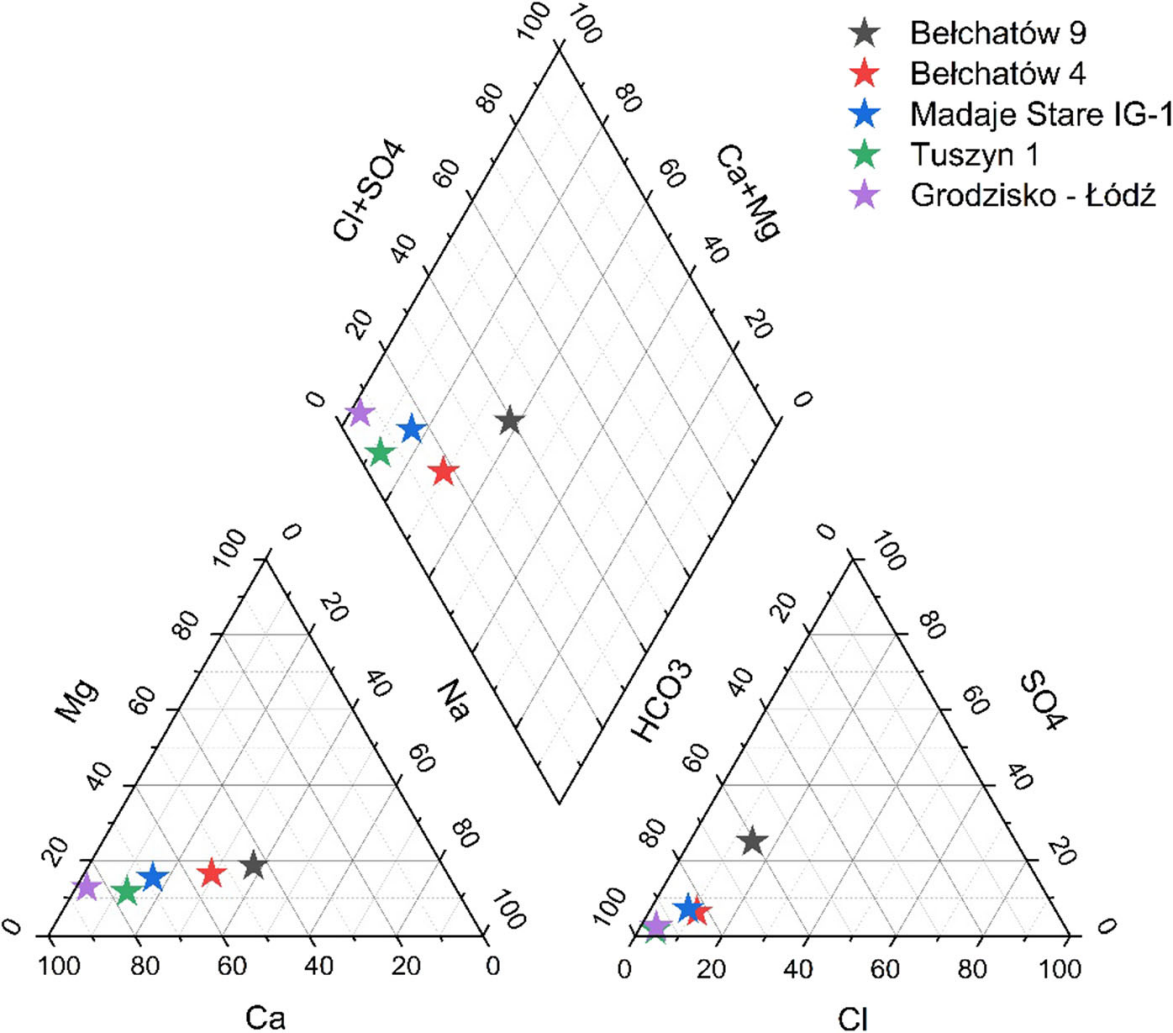
Piper diagram of waters from the Lower Cretaceous formations in the southern part of the Mogilno–Łódź Trough

The research area is located south of the Mogilno–Łódź Trough where lower values are observed. Waters from the southern part of the Trough have a low mineralization of a maximum of 0.5 g/L and are of Na–Ca–HCO_3_ or Ca–Na–HCO_3_ type (Hałaj & Kępińska, 2019). The chemical composition of the waters is presented in Fig. 4. The tops of the sampling interval are located at − 538 to − 1607 m a.s.l. There is some Fe occurrence from 0.2 to 4.5 mg/L in these waters, while none of them include I or Br ions (Hałaj & Kępińska, 2019).

Data from the area are very limited, and there is the only one well with the main ion’s analysis. The chemical composition of water from the Tuszyn 1 well located in the research area is shown in Fig. 4. The TDS of water is 0.2 g/L. The sampling interval is located at 760–765 m, and the measured flow rate is 3.24 m^3^/h (Górecki, 1990). The water is of Ca-HCO_3_ type.

Data for chemical equilibrium calculations were taken from a well, which is located ca. 2 km from the modelled area. Unlike wells from the area, the Grodzisko–Łódź well has a good chemical and hydrogeological research (Ziułkiewicz, 2003). Chemical composition of the water is shown in Fig. 4. Due to the lack of data, it was taken for further research as a representative for the chemical composition in the modelled area.

### Climatic data

**Fig. 5:**
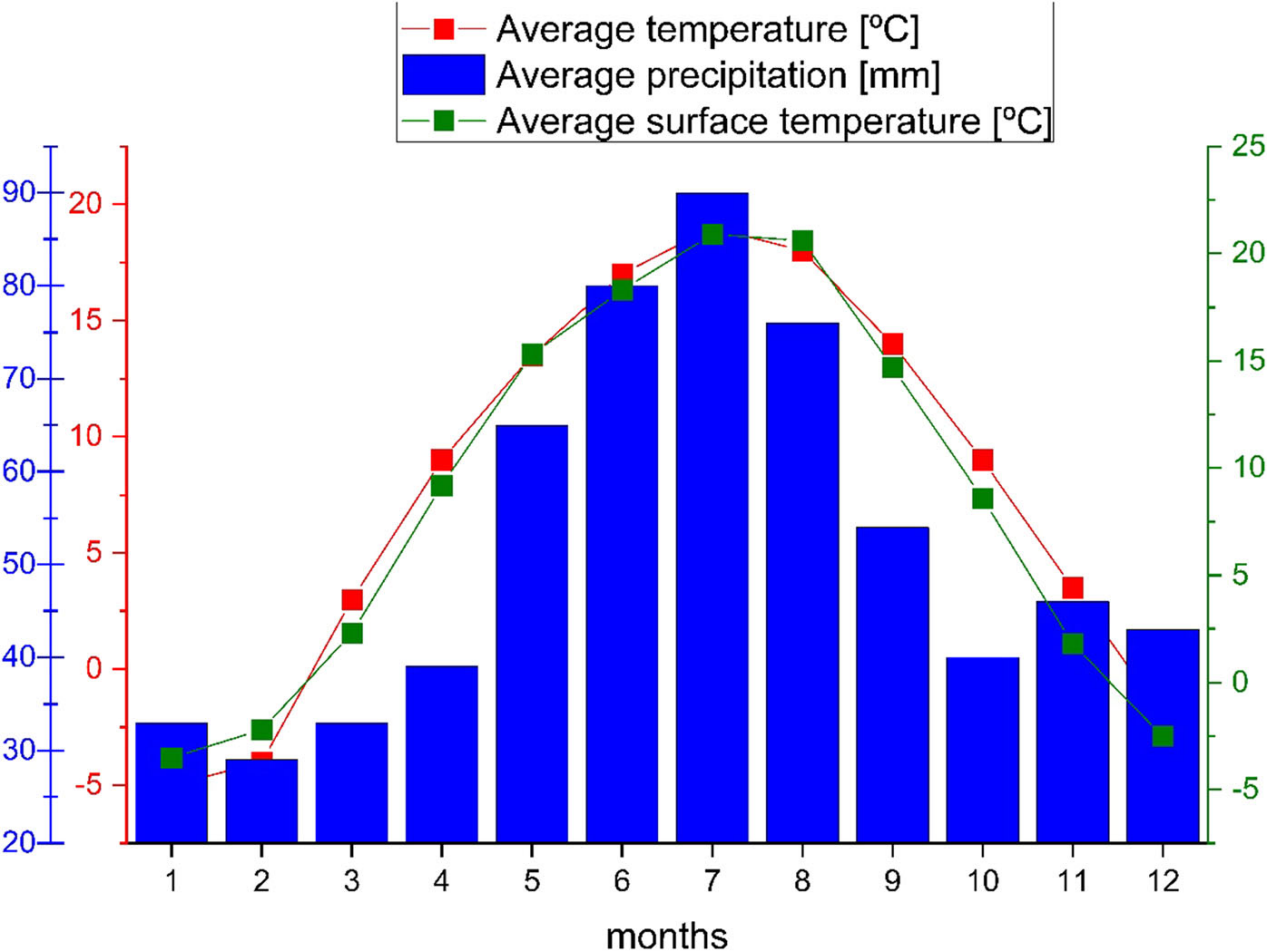
Average precipitation, air and surface temperature in Łódź, Poland (Climate-data.org)

The climatic conditions in the area are cold and temperate (Climate-data.org). The average air temperature in Łódź, the main city of the region, ranges from − 5 °C to 18 °C (Fig. 5). The average precipitation is from 30 to 81 mm. The Earth’s average surface temperature ranges from − 3.5 °C to 21 °C (Hałaj et al., 2020).

## Materials and methods

Presented results of research were obtained, using regional static models, customized to utilization in local scale. It means that the regional modelling results were just the input data for dynamical modelling, demanded considerable refinement and adjustment to data located within area of simulation. As all the research utilizes two incompatible software suits. It was important to establish specific workflow, enabling application of Petrel based static models for dynamic ATES simulations in FEFLOW. In the following paragraphs, authors present worked out methodology of such an adjustment, as well as the Feflow’s ATES simulations principles.

### Static model and the transition to the dynamic model

**Fig. 6:**
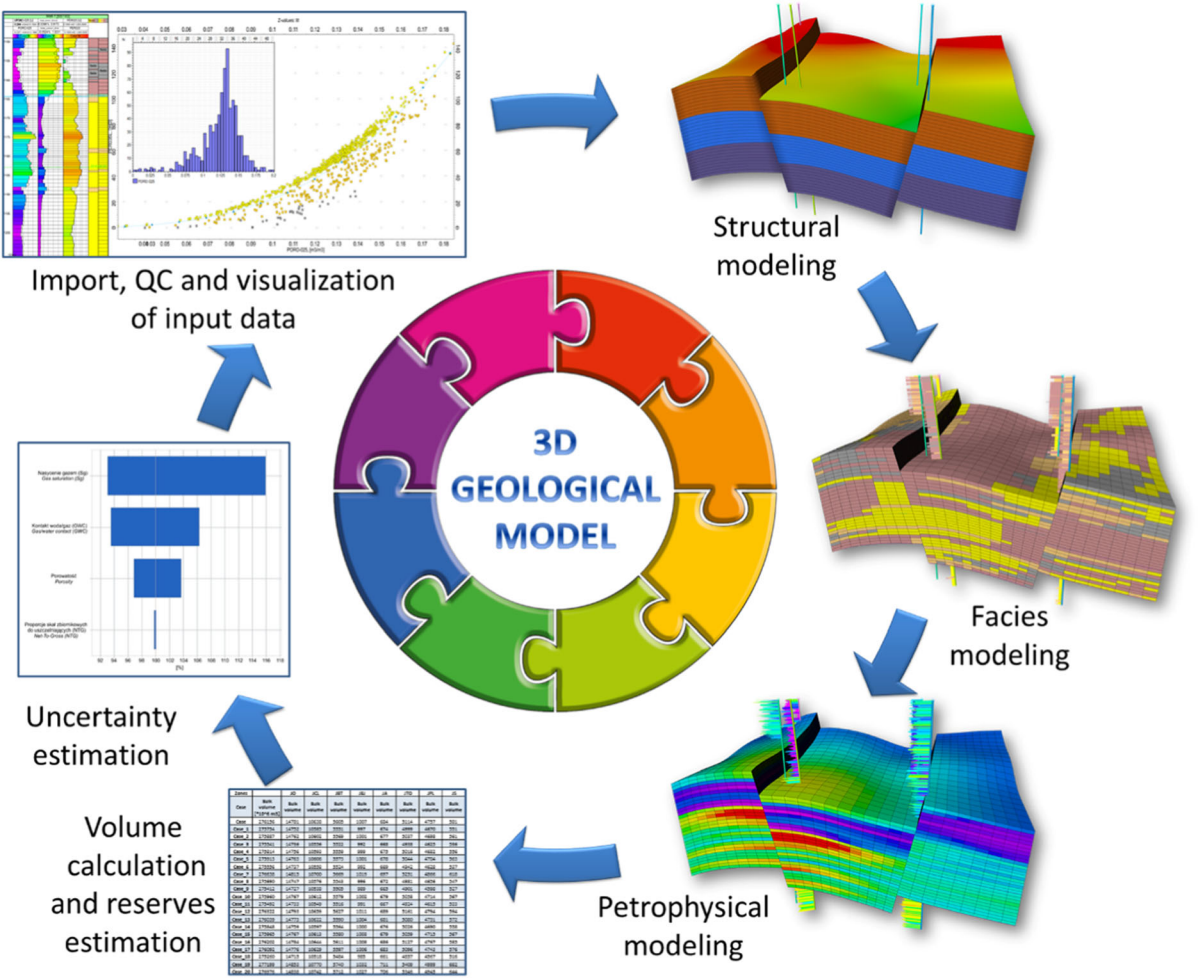
Static modelling workflow in petroleum and geothermic geology

Recent 3D static geomodels built for Research & Development purposes, in petroleum exploration, geothermic or energy storage fields, vary in scale from regional to local (Wygrala, 2014; Papiernik, 2014; 2017a, b; Papiernik & Michna, 2019). The models usually are very complex, handling large quantities of input data. A typical static modelling workflow (Fig. 6) comprises 6 main phases of modelling, starting from database building, through structural modelling, facies modelling, petrophysical modelling, up to volume and reserves calculations and risk assessment (e.g. Dubrule, 1998; Zakrevsky, 2011; Wachowicz-Pyzik et al., 2015; Vernik, 2016; Papiernik & Michna, 2019). Geological models used in the research demanded the completion of the four first phases.

**Fig. 7:**
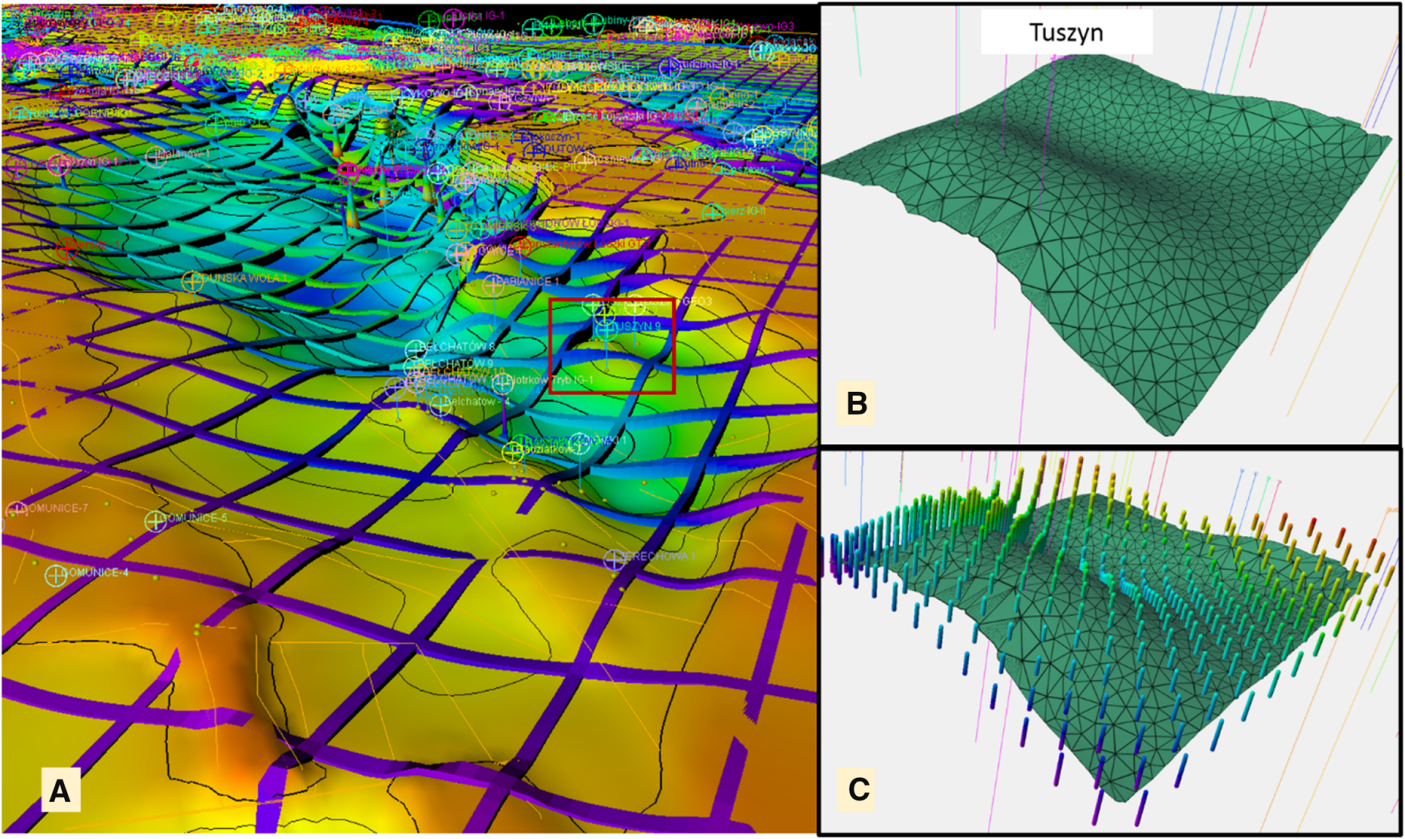
Adaptation of Petrel static model to the FEFLOW modelling requirements. **a** Regional structural and parametric Corner Point Grid of the Lower Cretaceous in the Mogilno–Łódz Trough (the Tuszyn area is marked with a red box); **b** local (Tuszyn Anticline) tetrahedral mesh of the Lower Cretaceous Top surface (based on (A)); **c** cells of a parametric temperature model (fences on panel (A)) converted into XYZG points used in FEFLOW modelling

The geological model of the Tuszyn Anticline was prepared with the use of Petrel. The basic workflows and input data used by authors to create geothermal 3D models of the Mogilno–Łódź Trough included models calculated with the use of the Corner Point Griding [CPG] method (Kępińska et al., 2017a, b, c). To utilize CPG-based geomodelling results for Feflow simulations, the regional CPG structural model (Fig. 7a) has to be transformed into the local structural framework model (Fig. 7b).

The dynamic ATES model uses the local structural and parametric framework, being the part of the basin scale parametric model of the temperature and effective porosity of the Lower Cretaceous reservoir. They were constructed as CPG grids, using the multiscale structural and parametric modelling workflows (Papiernik et al., 2015; Papiernik et al., 2016; Papiernik, 2017a, b; Papiernik & Michna, 2019. This allows for high-quality local geomodels to be obtained, even in regions poorly controlled with data. Such a case is the Tuszyn Anticline, where the low quantity and quality of input petrophysical and physical data disable the estimation of static modelling of a satisfying quality, using local data only.

### Dynamic model domain and boundary conditions

The simulations were implemented in Feflow. FEFLOW software belongs to the Finite Element Model dynamic simulators. The ATES system is modelled as a doublet of multilayer wells, which applies a pre-defined extraction or injection to a node or to a group of nodes along a well screen. The multilayer well boundary condition involves a method, which superimposes high-conductivity 1D tubular discrete features representing the well bore and well screens (Diersch, 2014).

The thermal energy storage system performance is simulated dynamically by an OpenLoop plug-in. There is no constant value of the injected/extracted temperature set. The plug-in adds a differential to the extraction temperature and sets the injection temperature as a temperature boundary condition at the injection well.

The geological assumption in the model is that the Lower Cretaceous formations are built of sandstones and constitute the aquifer while the top and bottom formations are mainly limestones of the Upper Cretaceous and the Upper Jurassic and constitute the low permeable confining.

The basic surfaces constraining the geometrical framework of the dynamic model at the Tuszyn Anticline—top and base on the Lower Cretaceous—were extracted from the regional model of Lower Cetaceous complex in the Moglilno–Łódź Trough (Kępińska et al., 2017a, b, c). The square part of this model was extracted and subsequently converted into formats applicable in Feflow (Fig. 7). Firstly, horizons from the CPG 3D grid were adopted as an input for the large scale, local structural model, created with the use of the structural framework procedure in Petrel. It allowed to estimate the surfaces of the Lower Cretaceous top and base in the form of the triangular irregular network (TIN, Fig. 7a, b). These TIN surfaces were next applied as an input for the geometrical Framework of the Feflow dynamic model.

The model domain was then subdivided into 86 layers in Feflow. The thickness of layers ranges from 0.1 to 135 m. The thickness of caprocks and the model bottom geometry (flat), which are less essential, were set in an arbitrary manner in order to assure different parameters of caprocks and may not necessarily reflect the individual geometry of the Upper Cretaceous and Upper Jurassic formations. The altitude of the top surface of the model was set to 200 m a. s. l., which reflect an average terrain height, while the bottom surface was set to − 2,000 m a. s. l. The dimensions of the model are 23.5 × 16.5 × 2.2 km. The depth of the Cretaceous layers is from 78 to 1375 m. The division in the sublayers of the model domain is to improve the quality of the numerical results. The Lower Cretaceous aquifer consists of 41 slices. The thickness of the layers gradually decreases in the horizontal contact zones of the aquifer. The primary horizontal mesh structure reflects the geological model structure, with the distance from nodes of 35 m. The mesh is suitably refined around the location of the wells, and the smallest distance is 0.15 m.

The petrophysical parameters for the dynamic model were approximated using the results of the regional static model. The model was generated with the use of the CPG method.

**Fig. 8:**
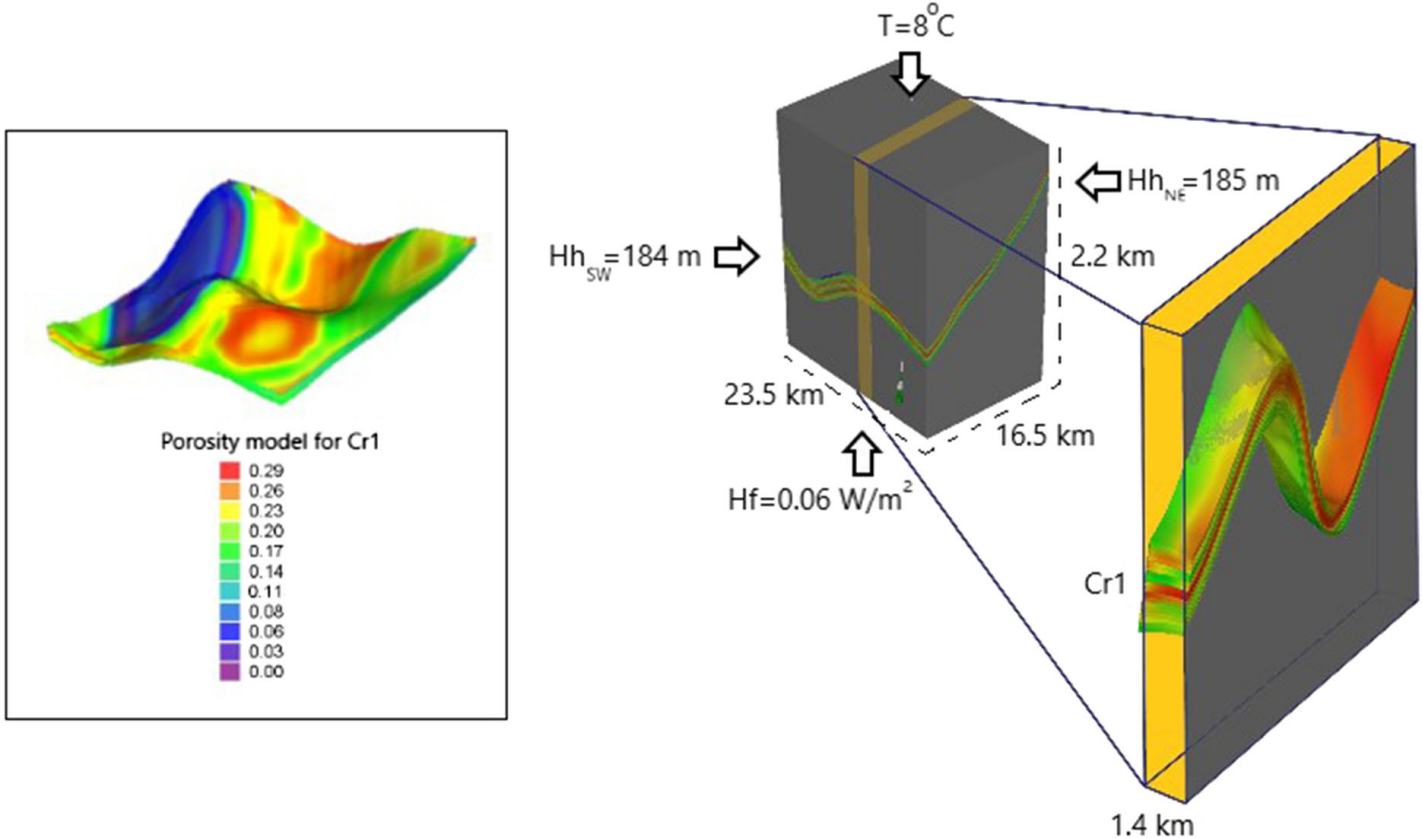
Model domain, thermal and flow boundary conditions and porosity model (Hh_SW_—hydraulic head at SW border, Hh_NE_—hydraulic head at NE border, T—temperature at the top surface, Hf—heat flow, Cr1—the Lower Cretaceous)

The hydraulic and thermal parameters are shown in Table 1. The porosity in the Lower Cretaceous formations was set according to geological static model. Porosity data (Fig. 3 and Fig. 8) for the geological static model was taken from the Archive of the Fossil Fuels Department at AGH. It varies from 0 to 0.29. The porosity in other layers was set as constant and it was 0.05. The thermal conductivity of the rocks was set for the Lower Cretaceous and the other layers as 2.8 and 2.5 J/m/s/K, respectively. These values were estimated from Plewa (1994) for both sandstone and limestone formations. The volumetric heat capacity of solid was adapted from Plewa (1994) and was set equal for all units. The volumetric heat capacity as well as longitudinal and transverse dispersivity were customized. These values were chosen after trial simulations because of reflecting the best thermal conditions from the static model.

**Table 1:** Hydraulic and thermal parameters implemented in the model. The parameters were assessed from Plewa (1994); Górecki (1996); Małecki et al. (2017)

	Caprock (Upper Cretaceous)	Aquifer (Lower Cretaceous)	Underburden (Upper Jurassic)
Dominant type of rocks	Limestones, not permeable	Sandstones, permeable	Limestones, not permeable
Hydraulic conductivity (Fluid flow) x, y, z	1 e-06 m/s	2 e-05 m/s	1 e-06 m/s
Porosity	0.05	0–0.29	0.05
Volumetric heat capacity of solid	2.2 MJ/m^3^/K	2.2 MJ/m^3^/K	2.2 MJ/m^3^/K
Thermal conductivity of solid	2.5 J/m/s/K	2.8 J/m/s/K	2.5 J/m/s/K
Thermal conductivity of fluid	0.65 J/m/s/K	0.65 J/m/s/K	0.65 J/m/s/K
Volumetric heat capacity of fluid	4.2 MJ/m^3^/K	4.2 MJ/m^3^/K	4.2 MJ/m^3^/K
Specific storage	0.0001 m^−1^	0.0001 m^−1^	0.0001 m^−1^
Longitudinal dispersivity	5 m	5 m	5 m
Transverse dispersivity	0.5 m	0.5 m	0.5 m

The average permeability of the Upper Cretaceous and Upper Jurassic aquifers is variable in rather small intervals and can be assessed as low (Górecki & Hajto, 2006). Better hydraulic conditions are observed in the Lower Cretaceous formations. Małecki et al. (2017) provide a hydraulic conductivity in the range from 5.8 e-04 to 7.75 e-05 m/s. Thus, the hydraulic conductivity of the Lower Cretaceous layers was set to 2 e-05 m/s. This value is comparable with values gained in field measurements for wells located in the area (Górecki, 1996). Other layers were considered as less conductive, and a value of 1 e-06 m/s was applied for each layer (Górecki & Hajto, 2006). Specific storage coefficient was adopted as default vales in Feflow. This value reflects realistic conditions well (Kuang, 2020).

The model is considered confined and fully saturated. The thermal and flow boundaries are summarized in Fig. 8. There is a shortage of hydrogeological data in the model area and some regional values were adapted. The horizontal recharge of the aquifer is considered from the north-east, where the infiltration area is located. Other potential recharge directions are the subject of debate between researchers (Rodzoch & Pazio-Urbanowicz, 2015), and they were neglected in this model.

The constant water heads of 185 m and 184 m were set as initial conditions in the NE and the SW borders, respectively. The values were assessed from a regional study (Rodzoch & Pazio-Urbanowicz, 2015). Hydraulic head initial conditions were then assigned by running the model in steady-state conditions.

The temperature distribution in the model is assumed to follow the geothermal gradient in the area, which is estimated at 2–2.2 °C/100 m (Hałaj & Kępińska, 2019). The base slice is defined as the boundary condition of the second kind. A heat flux of 0.06 W/m^2^ is applied according to Majorowicz et al. (2019). In the top slice, the boundary condition of the first kind with the temperature of 8 °C was set, which reflect the Earth’s average surface temperature in the Łódź area (Hałaj et al., 2020). The thermal regime of the model was obtained by providing the simulation in steady-state conditions.

The lack of high-quality petrophysical data in the Tuszyn Anticline enforced the application of *regional to local modelling workflows* used in the petroleum exploration practice (Dommisse et al., 2019; Papiernik & Michna, 2019). Such a downscaled regional model of the temperature was based on stabilized temperature logs from 222 wells and the porosity (PHI) logs from 257 wells, including the Tuszyn-2 well in the research area. The unpublished yet 3D temperature model was estimated for the entire Polish Lowlands. It uses the Kriging and is supported by the results of the experimental variogram and the 1D trend of the temperature variability with depth. This advanced Petrel modelling procedure required data analysis, comprising spatial distribution and trend analyses (in this case 1D trend) and 3D experimental variograms fitting. Application of this extended procedure considerably modifies typical kriging results. However, in 3D modelling of the highly non-stationary variables like temperature, it is indispensable from a methodological point of view (e.g. Dubrule, 2003).

The sections of the Petrel parametric models were converted into XYZG data format. They were used as very dense input data set for parametric model in the Feflow. It comprised 1,552,200 points that display a regular spatial distribution. This allowed the Feflow models to be populated evenly, using a simple kriging method.

**Fig. 9:**
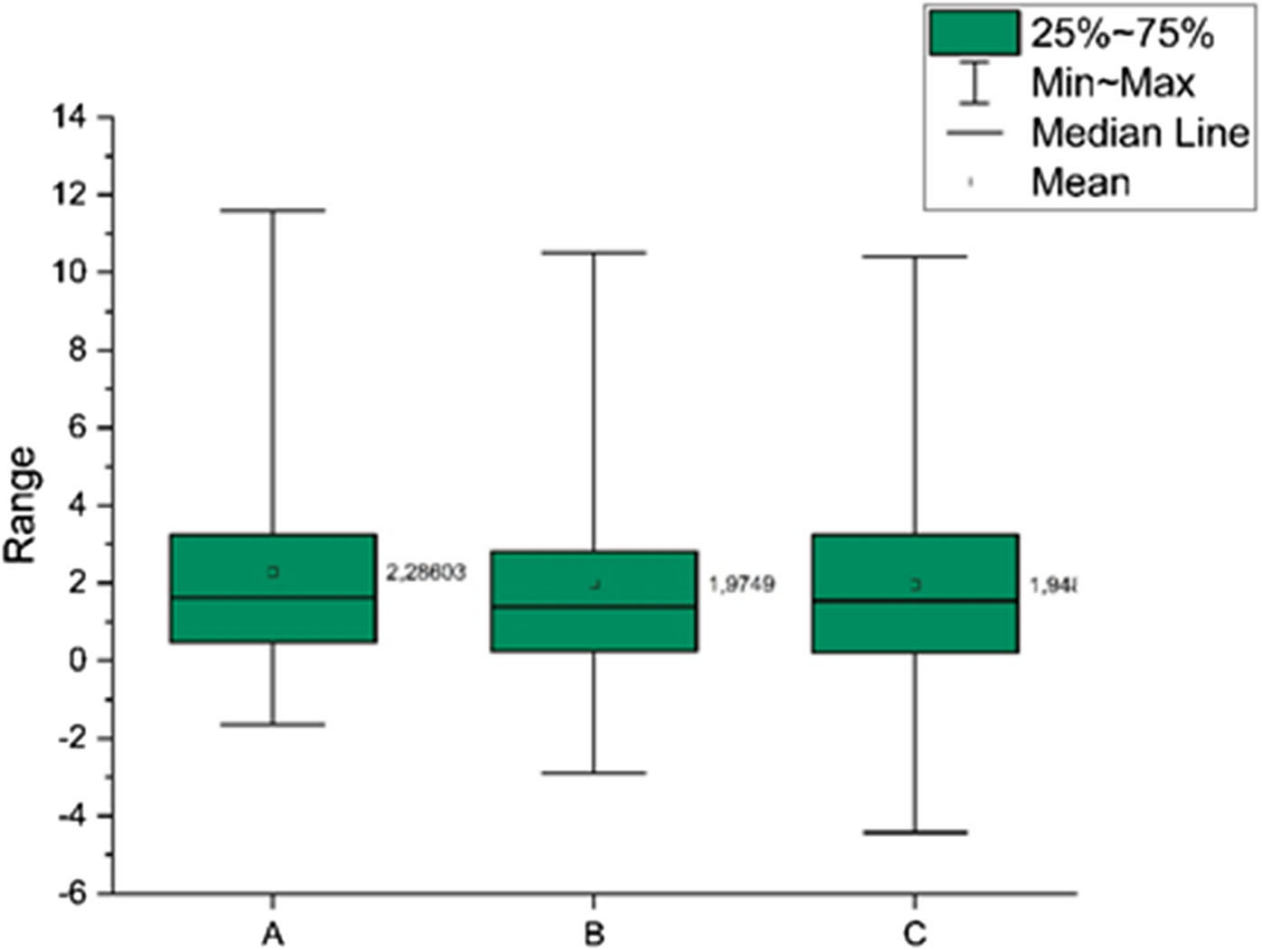
Temperature differences between Feflow and static model for the top (**a**), the middle (**b**) and the bottom (**c**) slices

The thermal model of the area was then validated by comparing it with the geological static model. The differences of the temperature were calculated for all points in 3 slices: the top, the bottom and the middle slice (Fig. 9). The assessed temperature difference in points is mostly less than 2.5 K, which was assumed as satisfactory. Using validated parameters and boundary conditions, the performance of ATES systems is then examined.

While the simulation is time-consuming, the model was then cut into a smaller one. The model was cut only in X-direction to 1.4 km and the direction of groundwater flow as well as geothermal gradient were kept the same while the Y and Z-directions were not cut (Fig. 8). The wells of 4 ATES systems were organized in this model. The injection and pumping wells have a diameter of 0.25 m and are placed in locations with different thermal regimes. The well doublets are always screened in the entire Lower Cretaceous layers. Doublets P-I 1–3 are of similar screening lengths (151–179 m). In the P-I 4 doublet located in the north-eastern part of the area, the well screen is shorter and is about 40 m long.

The well distance was chosen long enough to avoid an interference between wells in doublets. The undisturbed natural temperature of P-I 1–4 doublets ranges from 28.1 to 37.8 °C. Wherever there is a difference in temperature, K was used, while temperatures (values) were given in °C.

The life expectancy time interval for geothermal installations may be considered as 30 years. Therefore, modelled ATES system has been operating in cycling mode for 30 years, starting with heat injection. In ATES systems, temperature and flow rates are determined by heat consumption and supply. Time-varying values will generate time-consuming simulations and will extend the entire process. Therefore, some simplifications were introduced to fasten the quantity–quality description of the system. Each year is divided into 3 demonstrative, equal parts, simulating periods of accumulation, storage and the use of heat at equal load. The time series for each year starts with 121 days of heat injection, then the storage phase starts and heat extraction starts on the 243th day and lasts until the 365th day of the year.

A wide range of temperatures was considered, but the phase changing of ground water is not considered, while the range was set only for demonstrative purposes.

For each doublet, 4 temperature scenarios were applied. Injection wells were simulated to have temperature differentials of 10, 20, 30 and 40 K. Production wells have temperature differential of 20 K in each scenario. In future research, a realistic system work scheme for temperature and flow rate is planned. In production and injection wells, the flow rate stays constant and is 2,000 m^3^/d. Furthermore, in a comparative study a geothermal heating scenario was given by assuming only heat withdrawal for the last phase (starting from the 243th day of each year) with temperature differential of 20 K in a production well only. In geothermal heating scenario, all parameters and boundary conditions stayed the same as for ATES simulation.

### Thermal recovery ratio

To determine the efficiency of an ATES system in the Lower Cretaceous formation and the amount of recovered energy, the thermal recovery ratio can be calculated. The thermal recovery ratio η is defined as the ratio of energy extracted from the subsurface to the thermal energy injected in an operation cycle with respect to the natural temperature of the aquifer (Gao et al., 2019; Sommer et al., 2014).

Within this study, the thermal recovery ratio $\upeta$ was calculated according to:

1 $$\eta = \frac{{E_{e} }}{{E_{i} }} = \frac{{\mathop \smallint \nolimits_{{t_{0e} }}^{{t_{1e} }} v_{e} c_{w} \Delta T_{e} {\text{dt}}}}{{\mathop \smallint \nolimits_{{t_{0i} }}^{{t_{1i} }} v_{i} c_{w} \Delta T_{i} {\text{dt}}}}$$

where *E*—energy [J], *v*—water volume per time step [m^3^/s], *c*_*w*_—water heat capacity at constant pressure [J/ (m^3^ K)], $\Delta$ *T*—temperature difference [K], *t*_0_—time at the beginning of the process [s], *t*_1_—time at the end of the process [s], *e*—extraction phase and *i*—injection phase.

The thermal recovery ratio η was calculated for 30 years of operation in each doublet system. T_e_ and T_i_ values from each time step of the simulation were taken. Calculations were done for observation points located at the central point of the well in the middle of the well screening depth. While there is no heat injection, the thermal recovery ratio was not calculated for geothermal heating scenario.

### Hydrochemical calculations

The calculation of the saturation index of minerals was conducted in Phreeqc software v.3 (Parkhurst & Appelo, 2013) for minerals that are considered to occur in the research area. Temperatures used for calculation are assumed to be a possible reservoir temperature during the thermal storage and injection/extraction phases. The considered temperature range is from 0 °C to 150 °C. There is no literature available from which the mineral composition of the reservoir rock in the research area can be detailed. Si and Ca species, as dominant ones, were considered. Among them SiO_2_(a), chalcedony, anhydrite, aragonite and calcite may be the most common in sediments in the area. It is assumed that they may occur in the reservoir as main or accessory minerals. The precipitation of the species connected with changing circumstances and temperature increases can be one of the hazardous factors for the installation during the operational time providing a drop in the system’s efficiency.

### Dynamic modelling results and discussion

**Fig. 10:**
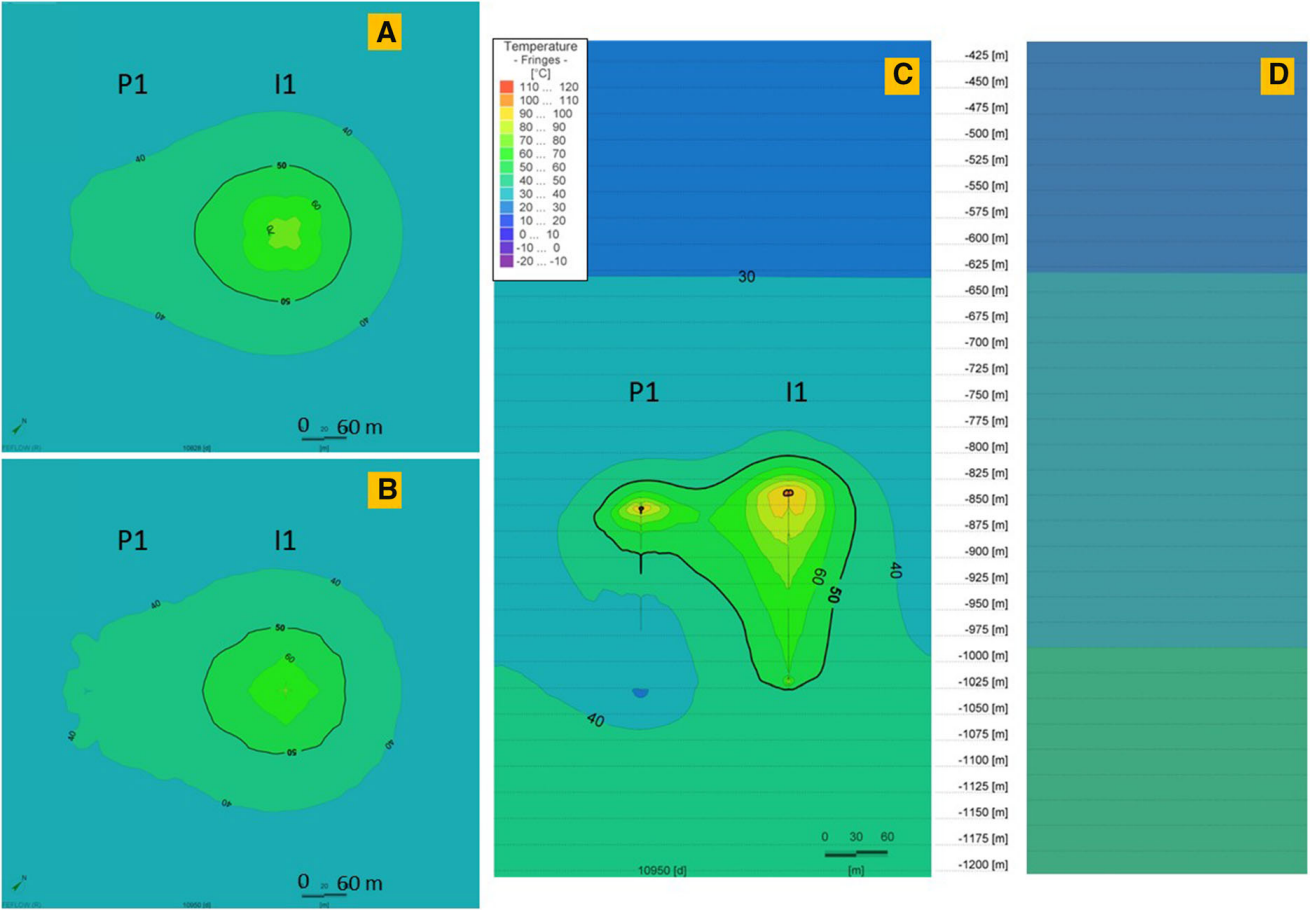
Thermal performance of the P-I 1 doublet for 30 K temperature differential on the end of the last, 30th storage phase (**a**) and at the end of the last year, after the 30th extraction phase (**b**) and the cross-sectional view at the end of the 30th extraction phase (**c**). **d** Initial temperature profile before wells operation (0 day). The legend is valid for all insets. P 1 and I 1 wells’ plain views located at depths 928.2 m and 917.5 m, respectively

**Fig. 11:**
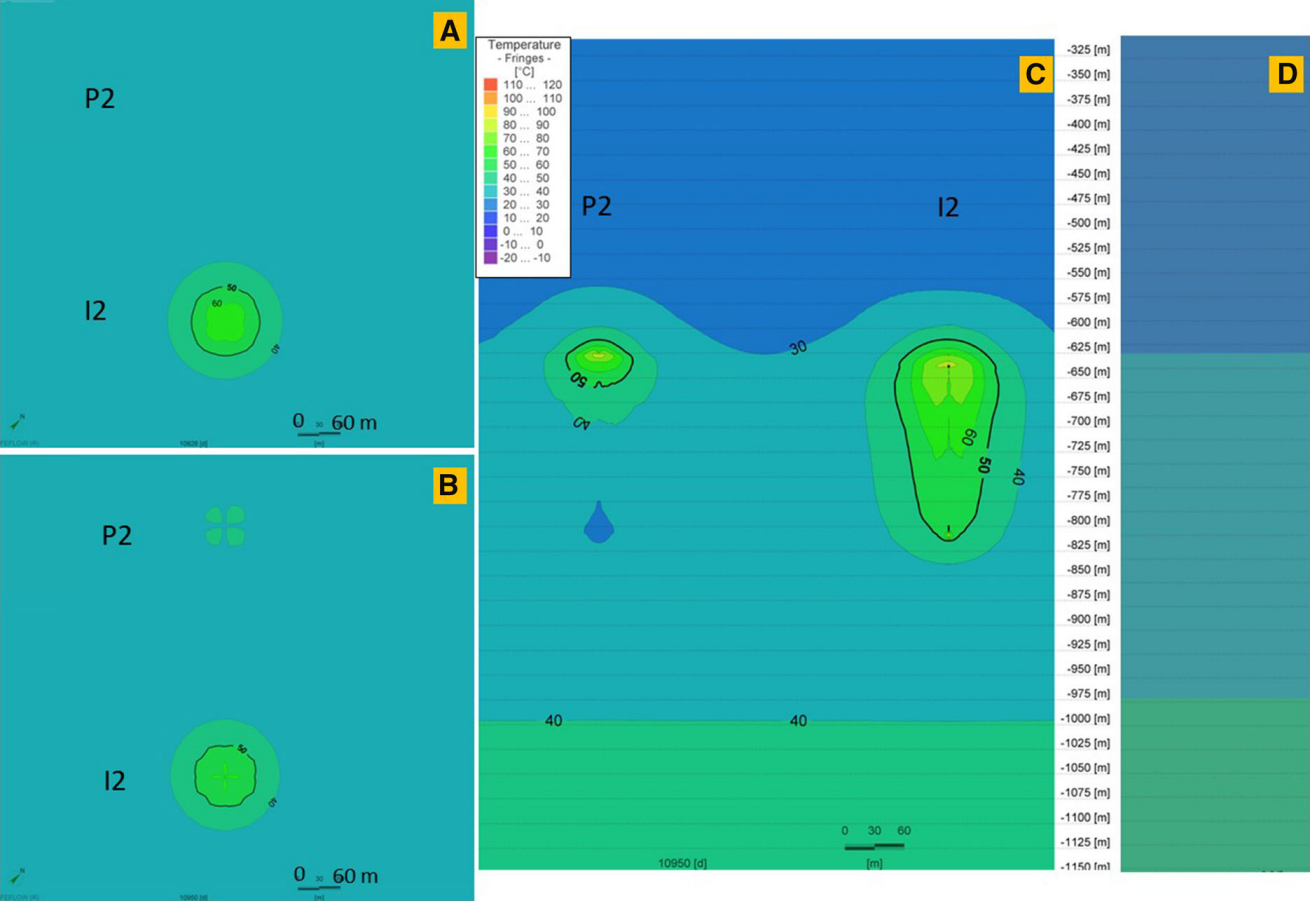
Thermal performance of the P-I 2 doublet for 30 K temperature differential on the end of the last, 30th storage phase (**a**) and at the end of the last year, after the 30th extraction phase (**b**) and the cross-sectional view at the end of the 30th extraction phase (**c**). **d** Initial temperature profile before wells operation (0 day). The legend is valid for all insets. P 2 and I 2 wells’ plain views located at depths 704.9 m and 711.9 m, respectively

**Fig. 12:**
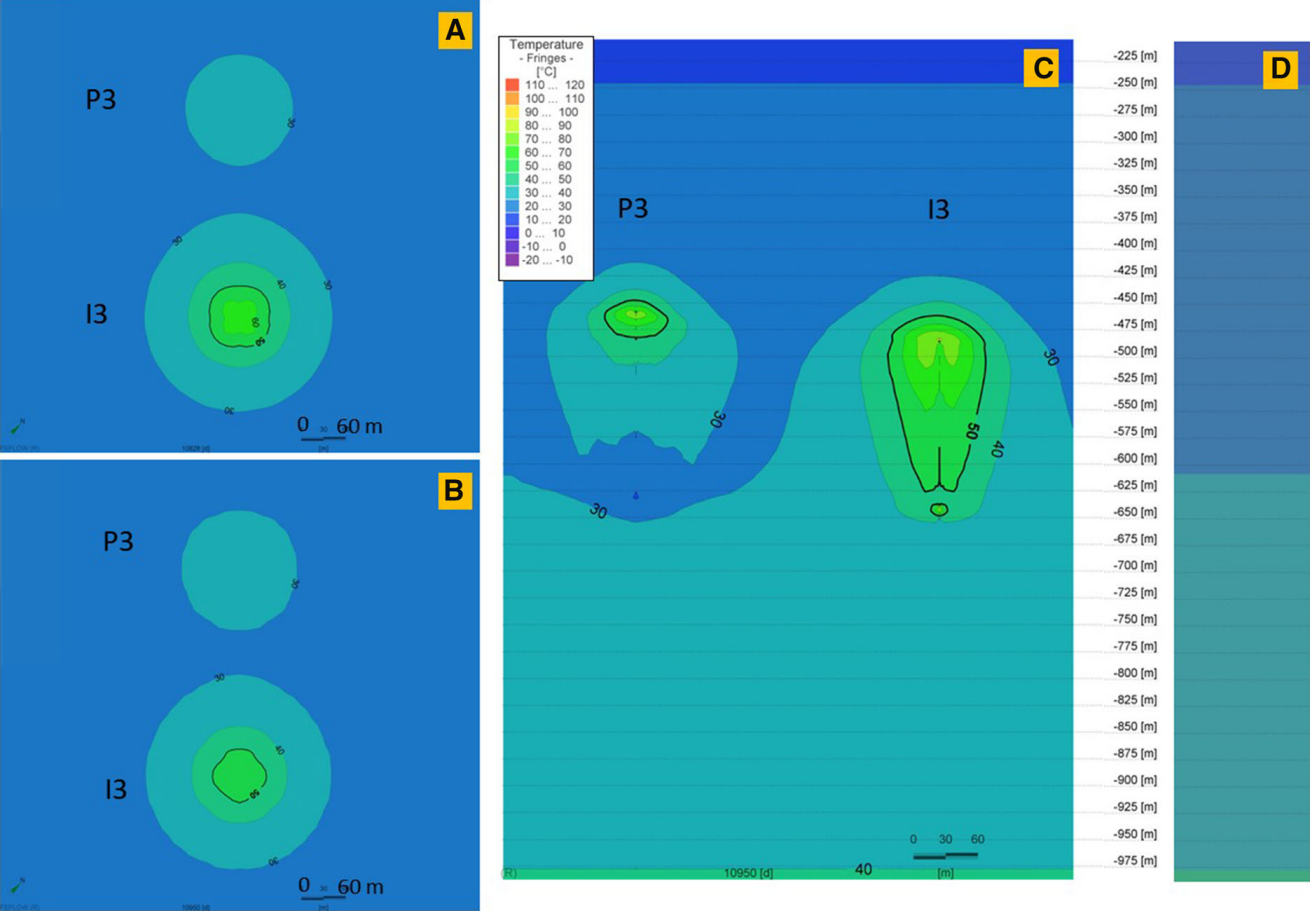
Thermal performance of the P-I 3 doublet for 30 K temperature differential on the end of the last, 30th storage phase (**a**) and at the end of the last year, after the 30th extraction phase (**b**) and the cross-sectional view at the end of the 30th extraction phase (**c**). **d** Initial temperature profile before wells operation (0 day). The legend is valid for all insets. P 3 and I 3 wells’ plain views located at depths 532.1 m and 552.9 m, respectively

**Fig. 13:**
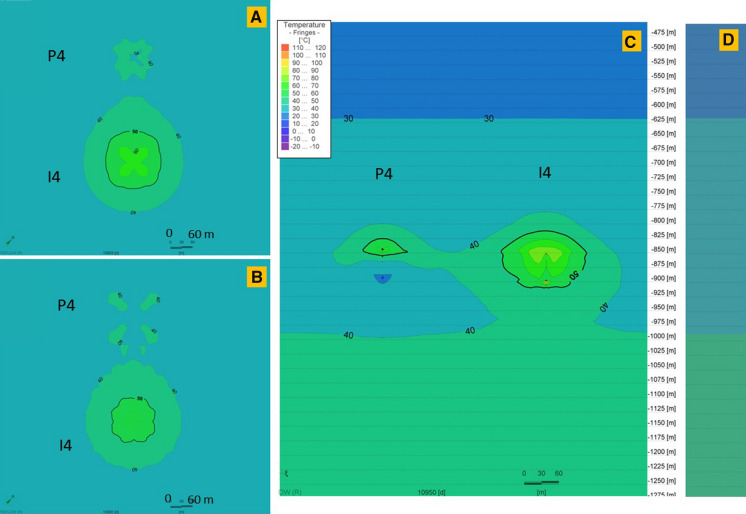
Thermal performance of the P-I 4 doublet for 30 K temperature differential on the end of the last, 30th storage phase (**a**) and at the end of the last year, after the 30th extraction phase (**b**) and the cross-sectional view at the end of the 30th extraction phase (**c**). **d** Initial temperature profile before wells operation (0 day). The legend is valid for all insets. P 4 and I 4 wells’ plain views located at depths 878.0 m and 886.9 m, respectively

Thermal performance of the storage process in P-I 1–4 doublets is shown in Figs. 10, 11, 12, 13. Each doublet is presented in plain view for 30 K temperature differential in 2 time steps: the 10,828th day–the end of the last storage phase and the 10,950th day (30 years) at the end of the last year, after the 30th extraction phase. The doublet view (Figs. 10 a, b; 11 a, b; 12 a, b; 13 a, b) is projected in the half of each well screen.

In the established model of energy storage, wells switch from extraction to injection and vice versa, according to the current phase. Production and injection wells were named after the first phase occurred in the well (until the 121th day). Therefore, the curves named as injection (I) have higher temperatures than the curves named as production curves (P).

The thermal performance of 30 K scenario, as one of the most promising, is shown in the main text. The thermal performance of doublets for 10 K, 20 K and 40 K scenarios are available in Supplementary Material **(Fig. A1–A4, Fig. B1–B4, Fig. C1–C4)**.

The well screen in P 1 well is 170 m, while in I 1 it is 174.4 m. Doublet wells are at a distance of 140 m. For 30 K temperature differential, a heat plume is observed around both wells in the P-I 1 doublet. In P 1, it is shorter than in I 1, where the temperature along the whole screening is higher than undisturbed natural temperature. In the upper part of the screen, a heat plume surrounds both wells together.

The well screen in P 2 well is 179.3 m, while in I 2 it is 164.8 m. Doublet wells are at distance of 352 m. For 30 K temperature differential, a heat plume is observed around both wells in the P-I 2 doublet. However, in the P 2 well the heat plume is declining in lower parts of the screen and has temperature similar to undisturbed natural temperature in the area or even lower.

The well screen in P 3 well is 166.1 m, while in I 3 it is 151.3 m. The doublet wells are at a distance of 282 m. For 30 K temperature differential, a heat plume is observed around both wells in the P-I 3 doublet.

**Fig. 14:**
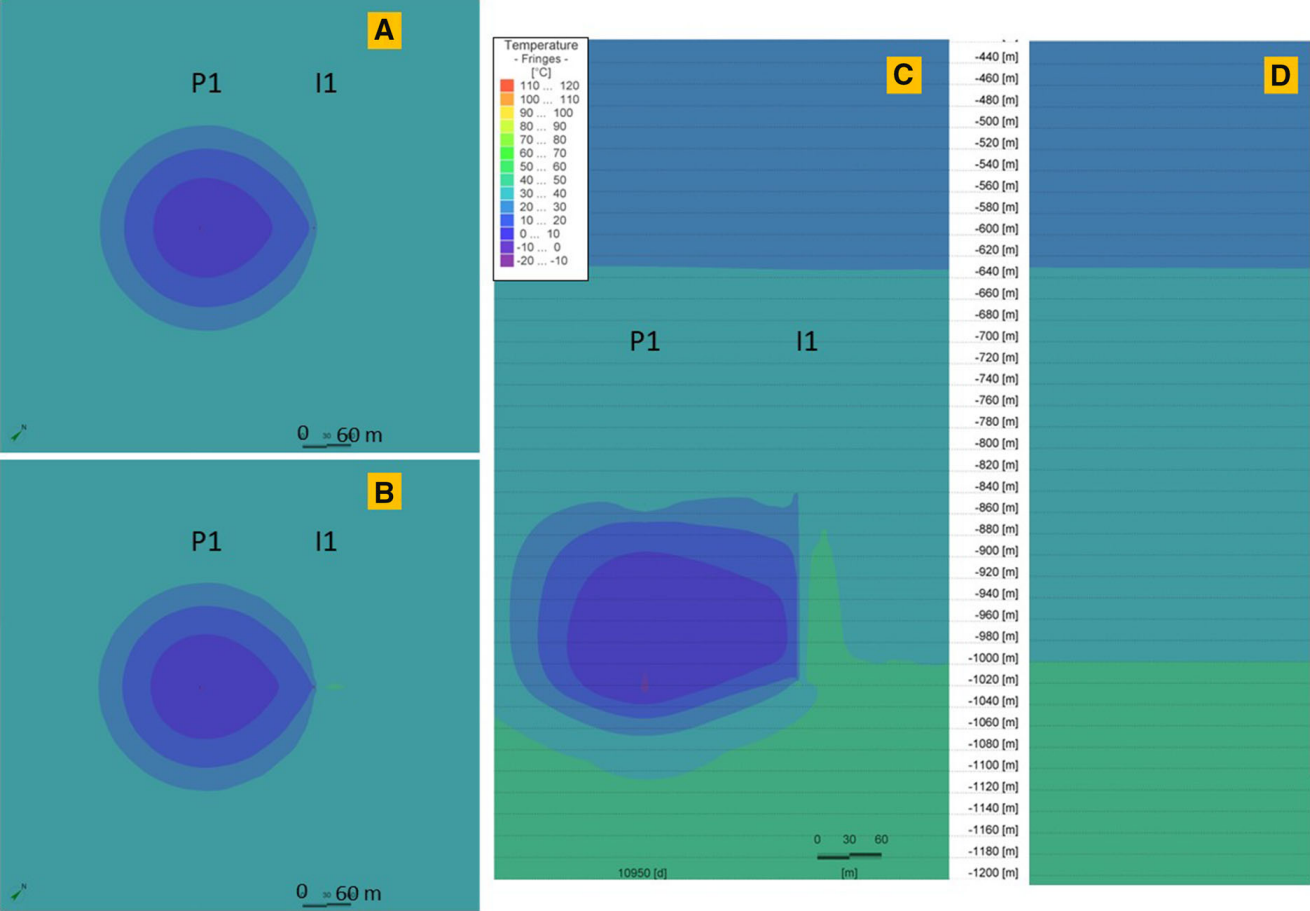
Thermal performance of the P-I 1 doublet for geothermal heating scenario on the end of the last, 30th storage phase (**a**) and at the end of the last year, after the 30th extraction phase (**b**) and the cross-sectional view at the end of the 30th extraction phase (**c**). **d** Initial temperature profile before wells operation (0 day). P 1 and I 1 wells’ plain views located at depths 928.2 m and 917.5 m, respectively

The thermal performance of doublets for geothermal heating scenario for P-I 1 doublet is shown in Fig. 14 for comparison. Thermal performance of other doublets is given in Supplementary Material **(Fig. D1-D3)**.

The well screen in P 4 well is only 42.4 m, while in I 4 it is 44.2 m. The doublet wells are at a distance of 284 m. For 30 K temperature differential, a heat plume is observed around both wells in the P-I 4 doublet. However, in P 4 well the heat plume is declining in the lower parts of the screen and has temperature similar to undisturbed natural temperature in the area or even lower.

**Fig. 15:**
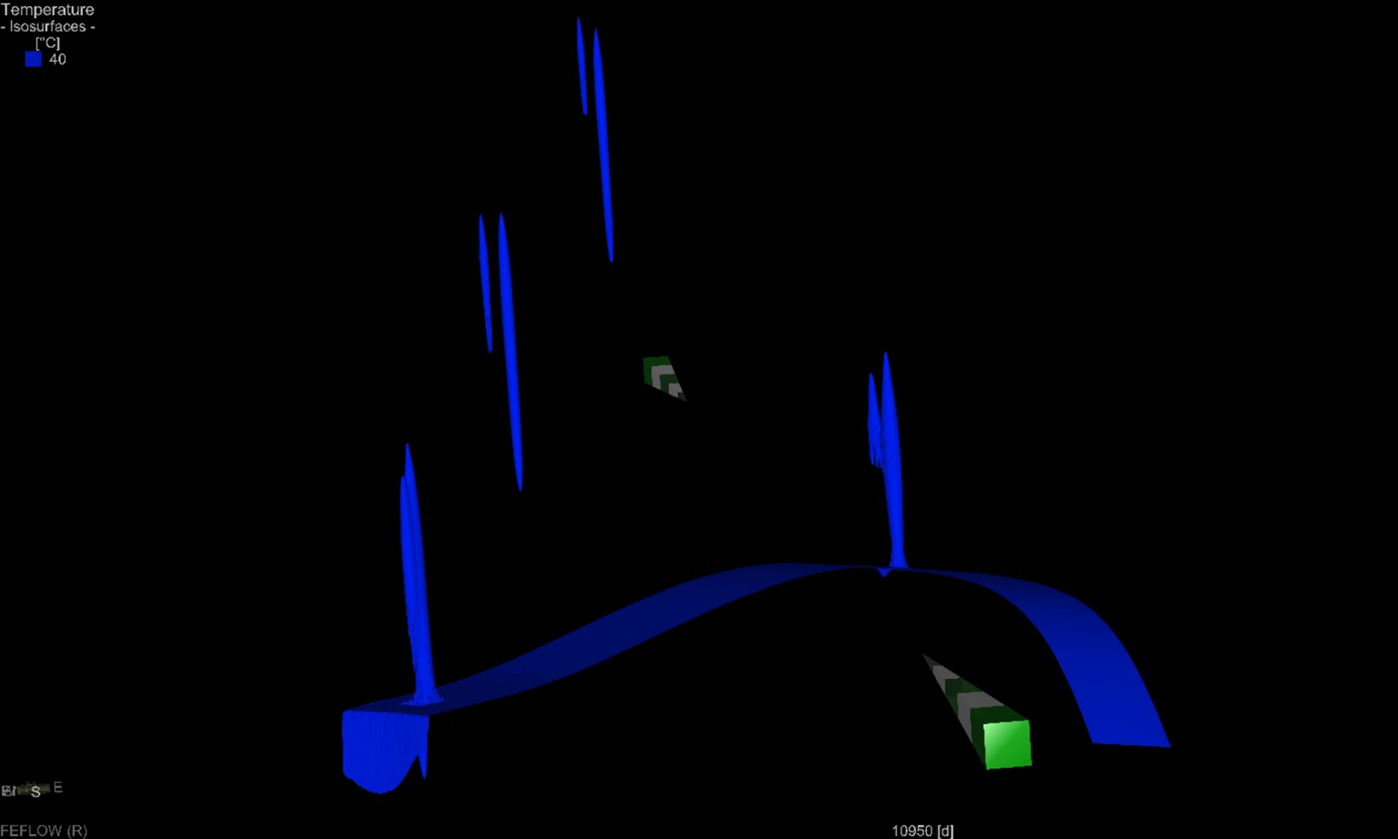
3D visualization of 40 °C isosurface for the ATES simulation within 30 K temperature differential scenario after 30 years

A 3D visualization of the heat plume at the end of the simulated period for the 30 K scenario is shown in Fig. 15. Other scenario visualizations are shown in Supplementary Materials **(Fig. E1–E3)**.

Unlike a typical geothermal system, in ATES systems the cold plume is partially balanced by the heat injection, which makes the system more efficient and should not allow the reservoir to cool down. In systems where the injected heat is greater than the extracted heat, the heat plume with a temperature higher than the undisturbed natural temperature occurs. The thermal plume is dependent on the heat amount injected to the system. It is the largest in wells with the highest temperature differential and short well screen.

The range of thermal impact on the surrounding rocks in the analysed wells is over several dozen metres. The heat plume’s range was measured within this study at half the distance of the well screen in the end of the 30^th^ year of operation (end of the extraction phase). In the P-I 1 doublet with a shorter well distance, both the heat (for 30 and 40 K) and cold plume (for 10 K) for both wells are aggregated form a compact plume, surrounding the doublet. For P-I 2, 3 and 4 wells, generate thermal plumes independently (**Fig A1–A4, Fig. B1–B4, Fig. C1–C4** in Supplementary material).

With a balanced injection and heat removal in the scenario for a 20 K temperature difference, the production well is surrounded by a cold plume and the injection well by a heat plume. In other cases, either both of the plumes are warm or cold.

Overheating and heat storage in the vicinity of the wells occur for temperature differential of 30 and 40 K. Round shape heat plumes with a temperature higher than the undisturbed natural temperature mostly occur. The ranges of the heat/cold plumes are given in Table 2. For geothermal heating scenario, only cold plumes occurred. They have the range bigger than in ATES scenarios and influence the injection wells.

**Table 2:** Thermal anomalies around ATES wells

Temperature difference	Heat/cold plume range definition	Thermal anomaly range [m]							
		P 1	I 1	P 2	I 2	P 3	I 3	P 4	I 4
10 °C	Isotherm of 25 °C for P wells and 35 °C for I wells	66	35	82	0	107	0	105	136
20 °C	Isotherm of 25 °C for P wells and 35 °C for I wells	29	80	47	54	78	0	73	39
30 °C	Isotherm of 40 °C for P and I wells	56	104	42	78	75	63	86	128
40 °C	Isotherm of 40 °C for P and I wells	100	117	68	100	60	90	134	151
Geothermal heating	Isotherm of 25 °C for P well	114	-	140	-	166	-	180	-

There is no heat plume for the 20 K and 10 K scenario. The temperatures obtained in this phase are much lower and rather cold plumes occurred. In wells I 2 and 3, the temperature is close to undisturbed natural temperature. In the case of 10 K temperature differential, there are even temperatures below 0 in the production wells P 1, 3, 4. In I 3, the undisturbed natural temperature does not exceed 30 °C, so the range of the heat plume is limited.

When there is a surplus of heat injected into the well, the temperature around the injection (warm) well increases. During the storage phases, when the heat injection stops, the temperature in the “warm well” decreases, but is higher than undisturbed natural temperature and enables to start the production of water with a higher temperature.

During the extraction phase, the temperature from the “warm well” decreases according to the temperature differential. In the “cold well”, where the cooled water is injected to the system during that phase, the temperature decreases simultaneously. Nevertheless, the temperature of the reservoir increases over time for 30 and 40 K temperature differentials. After the end of the last extraction phase, the increase in the temperature in P-I 1, 2, 3 doublets is of a max. 24.9 °C and 49 °C, respectively, in comparison with the initial, undisturbed natural temperature. A slight decrease in the final temperature is only noted in the P-I 4 doublet with the highest undisturbed natural temperature. The temperature drops there by 1.2 K in the P-4 well.

For 20 K temperature differential scenario in P-I 1–4, temperature drops in the course of 30 years. In the last years, temperature has stabilized and its fluctuations are smaller. For this scenario in P-I 1–4 doublets, temperature has decreased by a max. of 28.6 °C, reaching the lowest temperatures.

**Fig. 16:**
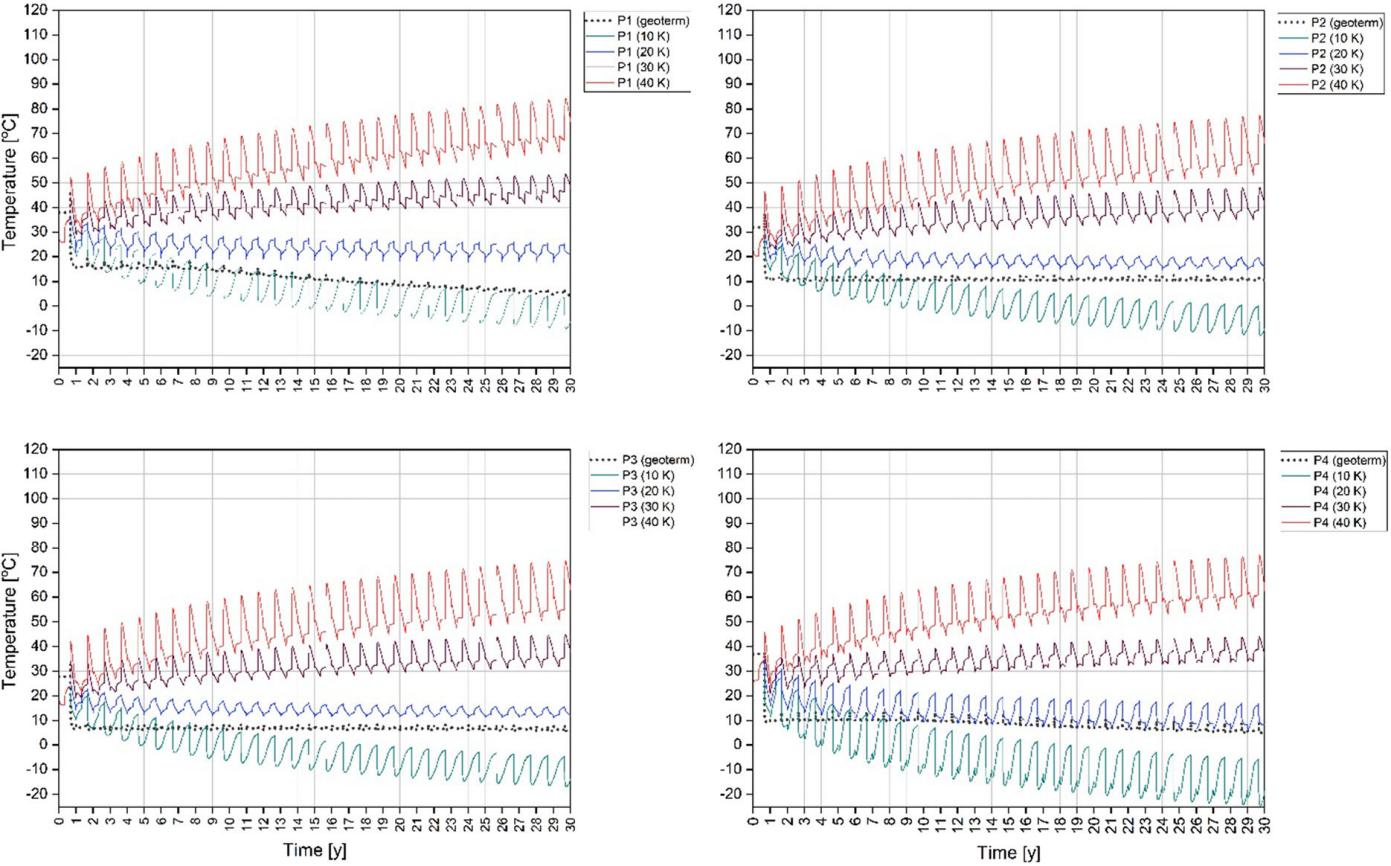
Temperature changes around P 1–4 wells at a depth of a half of the length of the well screen during 30 years of operation for 10 K, 20 K, 30 K, 40 K temperature differential (solid lines) and geothermal scenario (dotted lines). Observation points are located in the centre of well screen

**Fig. 17:**
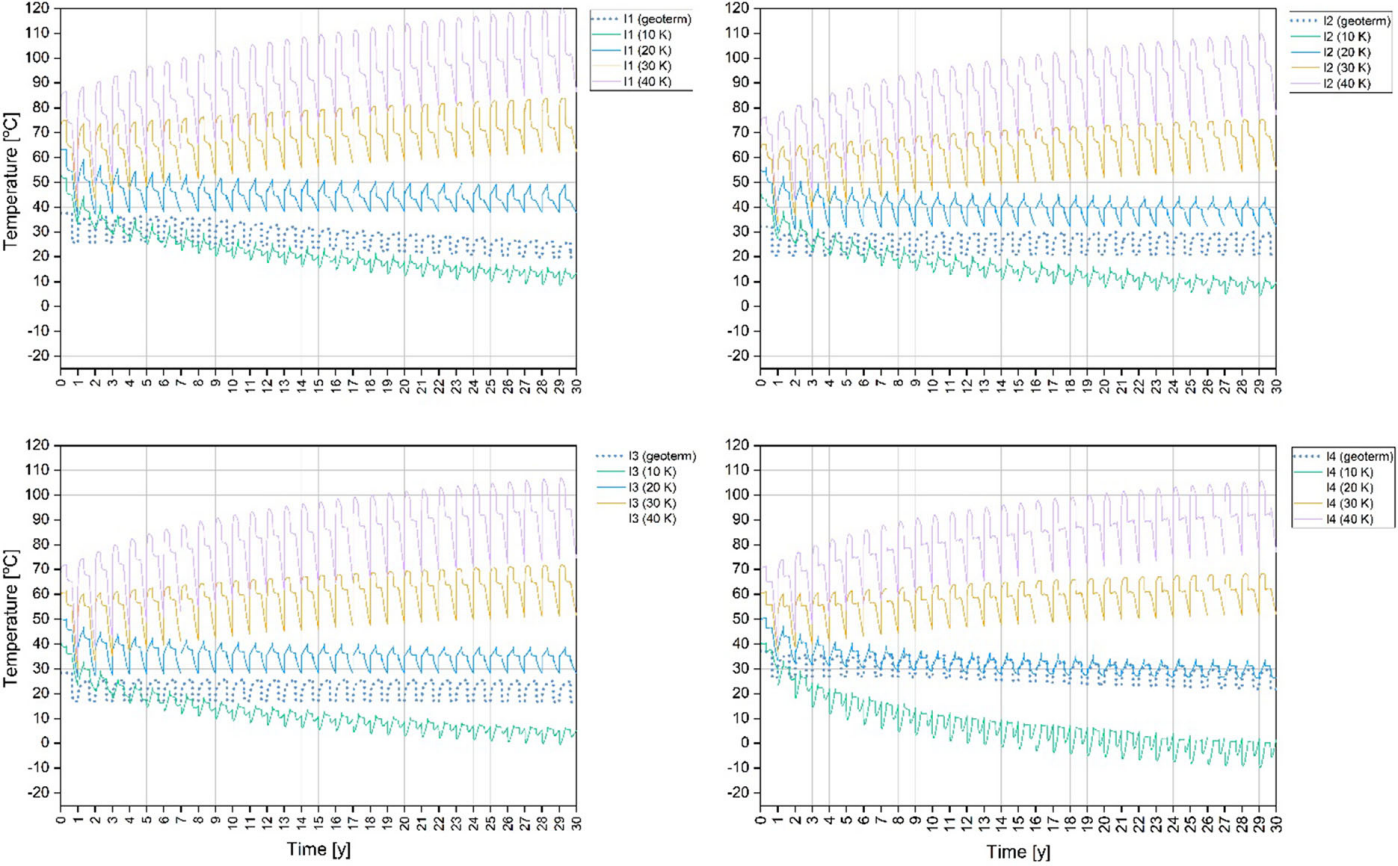
Temperature changes around I 1–4 wells at a depth of a half of the length of the well screen during 30 years of operation for 10 K, 20 K, 30 K, 40 K temperature differential (solid lines) and geothermal scenario (dotted lines). Observation points are located in the centre of well screen

For 10 K temperature differential scenario, the temperature has decreased over time because the heat injected to the system is not balanced with the extracted heat. After 30 years, the temperature in P-I 1, 2, 3 and 4 doublets dropped by a max. of 55.4 °C reaching values below 0 °C. An average temperature below 0 °C is shown for P 1–4 wells from the 3^th^ to 13^th^ year of operation. In I 3 and 4 wells, the temperature reaches values below 0 °C since 29^th^ and 13^th^ year of operation. Changes in the water phases were not considered, but it was shown that the temperature drop is significant excluding this scenario. Changes in temperature for observation points in wells are provided in Figs. 16 and 17. The higher the temperature differential for the injected water, the reservoir temperature increase is more significant. The highest temperature is observed in the P-I 1 doublet. This doublet has the highest undisturbed natural temperature and the shortest distance between wells.

The simulation results were also compared with the results of the geothermal heating application without heat injection in the comparative study. In the heating application, where the water withdrawal is only in the production wells, the temperature level in both the production and injection wells is rather stable with only small decreases in the 30-year operation time for the P-I 2 and 3 doublets. Simultaneously, in the 20 K scenario, I 4 curve starts to coincide with the geothermal curve from the 8th year, while in the remaining I 1, 2, 3, curves operational temperature is higher than the geothermal one. In the P-I 1 and 4 doublets, the geothermal scenario temperature drop down starts from the 9th year of operation and is max. 18 K lower after 30 years than the starting temperature. At the same time, the temperature from curves in the 20 K scenario is increasing.

Even if the heat withdrawal is not balanced by injection, the temperature in the well is balanced by the reservoir during stopping phases. Namely, the reservoir temperature has time to get back to the natural values. However, the temperature obtained in the geothermal application is considered as too low for direct heating purposes. It is a max. of 21 °C. The only option to begin using the heating system in that reservoir are heat pumps. On the opposite ATES done with 30 K and 40 K temperature differential, the scenario can ensure a higher temperature for the direct applications for waters.

In the 10 K scenario where a thermal balance is negative and a temperature differential for injection is set at 10 K, while the temperature differential for extraction stays at 20 K, the temperature in the wells decreases significantly. The reservoir is not able to balance the temperature due to wells switching between injection and production during the operation period. Therefore, switching between injection and production (warm and cold wells) during thermal energy storage is not sufficient, when not disposing of the proper temperature for injection. The obtained temperatures are also too low for direct heating purposes. Additionally, for the P-I 4 doublet, the decrease in the temperature is the most significant, despite having an undisturbed natural temperature similar to the P-I 1 doublet. This can be influenced by a shorter well screen in the P-I 4 doublet and a larger thermal plume range, which makes it slower in thermal restoration. A shorter distance favours an increase in temperature, but with a shortage of heat (10 K) the temperature drops faster. An overly low temperature difference can result in a low reservoir temperature. Hence, the opposite of expected scenario for thermal storage.

A low undisturbed natural temperature as in the research area means that in order to obtain the proper temperature, injecting heat with an appropriately high-temperature differential, which causes large increases and decreases in temperature in the wells, is required. The lower the temperature differential, the lower the fluctuations, but the thermal storage and withdrawal effect is worse.

Some remarks should be done for the additional heat needed to be injected to the system in ATES. For the research area, the best would be sources where a temperature differential high enough is guaranteed. One of the high-temperature heat sources can be large installation of solar collectors or waste heat from cooling tower of a power plants. In fact, a power station is in the area, but it is located ca. 35 km from the research site, which seems to be inefficient in the heat transfer to the ATES systems. However, a similar study can be conducted for the reservoirs nearer to the power plant by applying a field investigation in order to get more details.

### Thermal recovery ratio

**Fig. 18:**
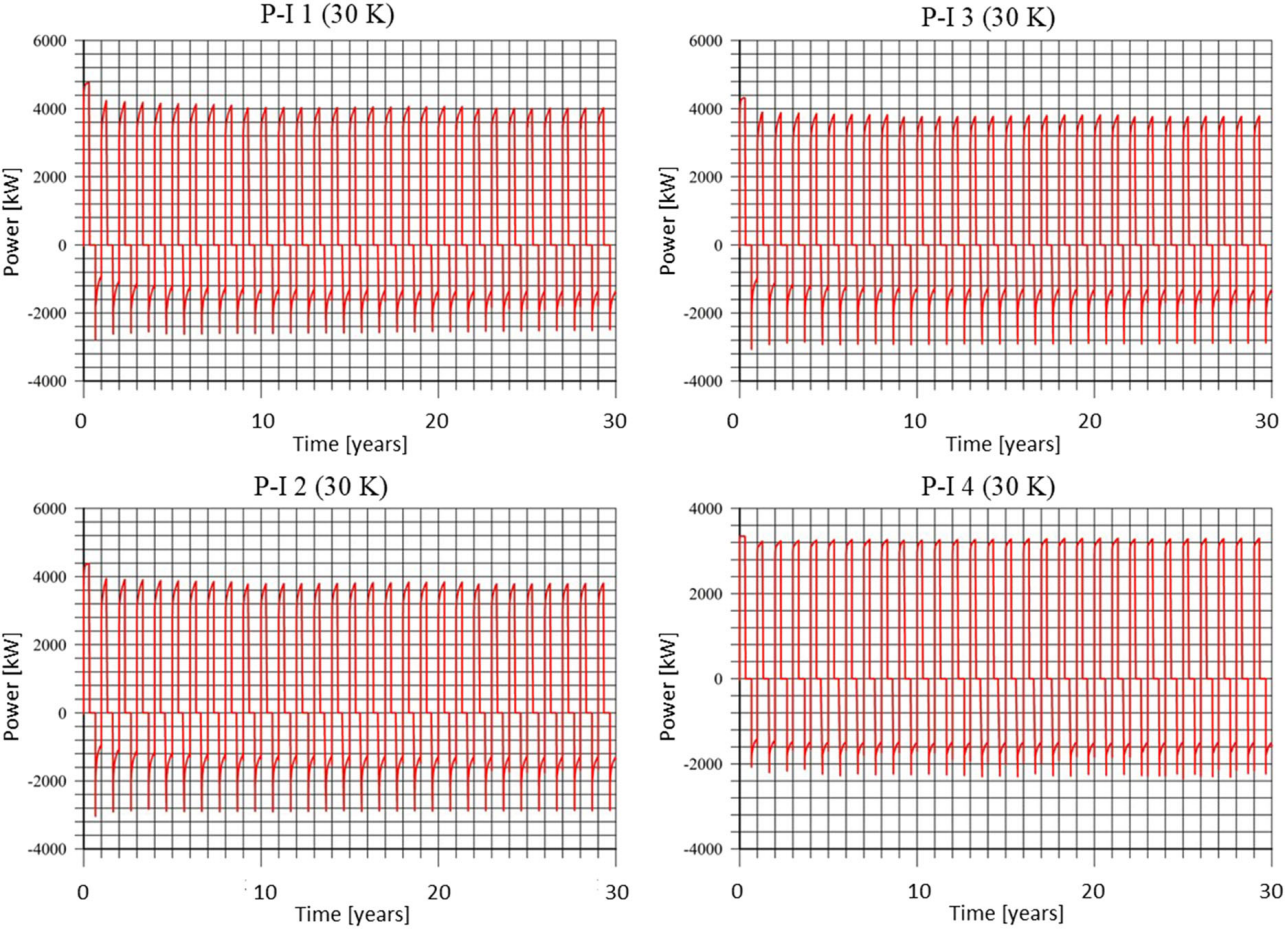
Thermal power for the P-I 1–4 doublets in the 30 years of operation for the 30 K temperature differential asset. Each year consists of 3 phases: injection until the 121st day, storage until the 243rd day and extraction until the 365th day

The thermal power for doublets P-I 1–4 for 30 K scenario are shown in Fig. 18. Thermal power can be negative or positive. Negative values are related to thermal energy extraction, while the positive for the thermal energy accumulation in the reservoir. Other power graphs are available in Supplementary Material **(Fig. F1-F3)**.

The thermal recovery ratio η calculated according to (1) for all the wells is from 0.34 to 0.94 (Table 3). Obtained thermal recovery ratios are of moderate values. The balance of injection and extraction temperatures depends on the scenario. Thermal recovery ratio values are the highest in the 20 K scenario, where an injection and extraction temperatures are set equal. In this scenario, temperature of water is, however, low, which is undesired in the direct uses of heat obtained from the reservoir. On the other hand, the 40 K scenario has the highest waters temperature, but low thermal recovery ratio.

**Table 3:** Thermal recovery ratio decently on temperature difference for assumed doublets, values estimated within 30 years of exploitation

Doublet no.	Temperature difference			
	$\Delta$t inj. = 10 K $\Delta$t ext. = 20 K	$\Delta$t inj. = 20 K $\Delta$t ext. = 20 K	$\Delta$t inj. = 30 K $\Delta$t ext. = 20 K	$\Delta$t inj. = 40 K $\Delta$t ext. = 20 K
1	0.64	0.73	0.47	0.34
2	0.46	0.77	0.49	0.36
3	0.36	0.78	0.50	0.37
4	0.34	0.94	0.58	0.42

*inj*. injection; *ext*. extraction

### Saturation Index (SI)

The injection, storage and extraction cyclic phases make large differences in reservoir temperature, which may then influence changes of the system geochemistry. To assess the possibility of precipitation of several minerals, saturation indexes (SI) were calculated for water from Grodzisko–Łódź well.

**Fig. 19:**
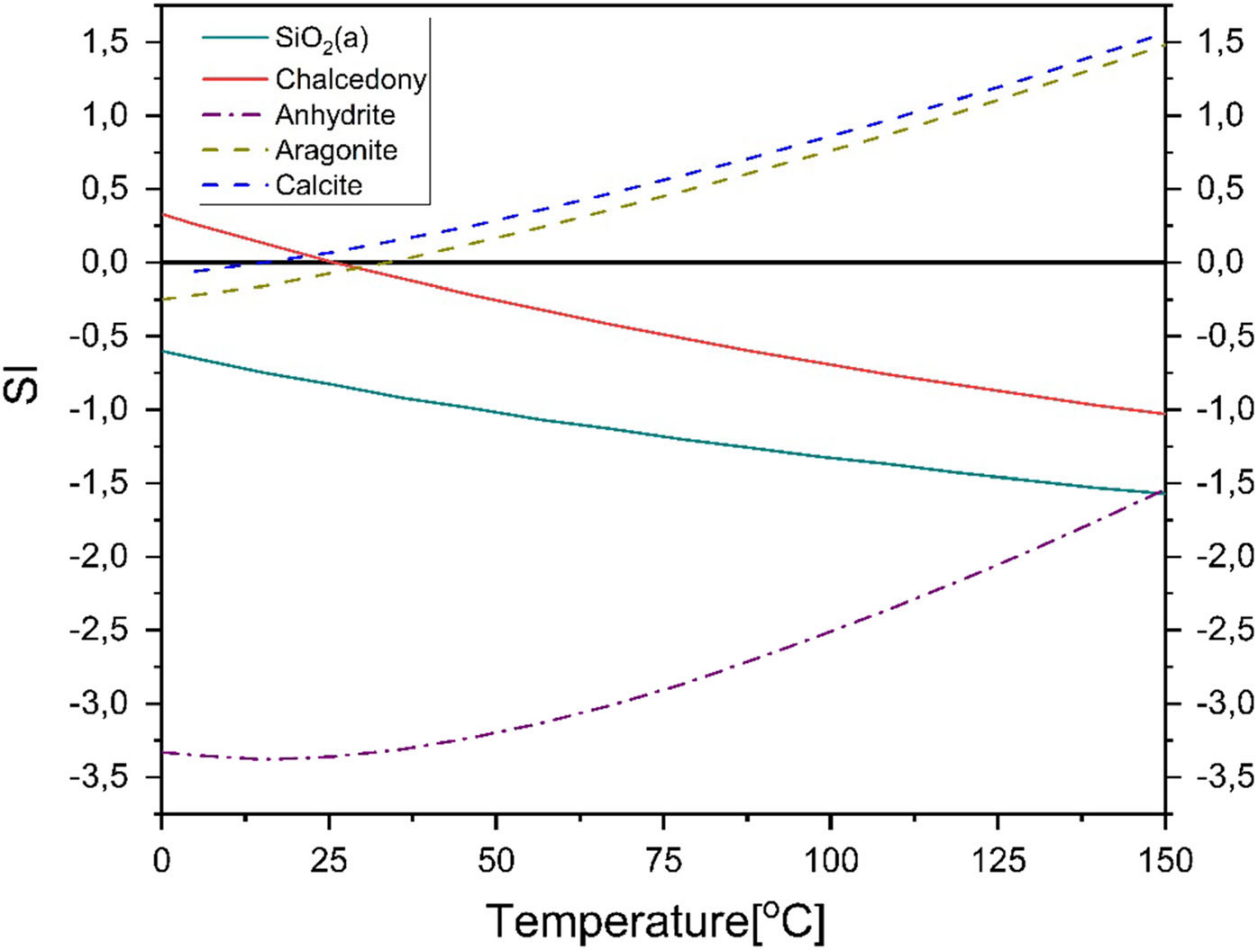
Saturation index (SI) for a chosen species in a different reservoir temperature for reference water from the research area

The saturation index calculations are limited to main and accessories minerals of the Lower Cretaceous. The most attention is given to Si and Ca minerals, while sandstones are the main rock type for the Lower Cretaceous formations and Ca minerals can also occur as main or accessory minerals. SI of SiO_2_(a), chalcedony, anhydrite, aragonite and calcite species are provided in Fig. 19.

SiO_2_(a) and chalcedony have a tendency towards undersaturation with an increase in temperature. Precipitation and clogging caused by SiO_2_(a) are potentially not hazardous within the entire temperature range, while chalcedony is not potentially hazardous at a temperature over 25.7 °C. It may precipitate below this temperature. SiO_2_(a) and anhydrite are undersaturated within the entire range of reservoir temperature changes, and it may be assumed that these minerals are not going to precipitate during the production, injection nor storage process. Some precipitating potential is also showed by CaCO_3_ minerals: aragonite and calcite with SI values > 0 starting from temperature of 36 and 15.4 °C, respectively. Even the SI values for anhydrite increase with the temperature, it has still negative values during the entire temperature range and minimal precipitation hazard.

The most hazardous minerals for the ATES installation in the research area in terms of minerals precipitation are aragonite and calcite. The occurrence of these minerals in the reservoir rocks is potentially hazardous for higher temperatures obtained in such systems.

The calculated SI results are comparable with thermodynamic modelling of several geothermal wells in the Lower Cretaceous in the Łódź Trough obtained by Wiktorowicz (2014).

Changing reservoir temperatures in a range considered for ATES provides changes in thermodynamic equilibrium. The higher the temperature injected to the reservoir, the higher the risk of clogging caused by aragonite and calcite. On the other hand, intensive thermal harvesting causing the low temperatures occurrence may provide a risk of SiO_2_ precipitation, while aragonite and calcite are undersaturated.

The provided geochemical modelling of SI index gives an initial assessment tool for the expected impact of the ATES system on the reservoir and the infrastructure. It may allow decisions to be taken to minimize the precipitation impact on an ATES installation and plan the scope of pre-treatment during the system operation.

## Conclusion

A determination of the possible performance of the ATES systems in the southern part of the Mogilno–Łódź Trough was obtained by multidisciplinary approach. It is the first step in assessing the possibility of the system implementation in a specific reservoir.

ATES can be used for increasing the Lower Cretaceous reservoir temperature and making groundwater an efficient source of energy, even in places where the geothermal potential for direct purposes is low. Considering the depth of the Lower Cretaceous aquifer and its undisturbed natural temperature in research area, maintaining a high temperature difference for heat injection and storage is essential. ATES systems for this kind of reservoir must be designed to maintain balanced heating/injection performance. Otherwise, the system will not be efficient, using temperatures lower than those in a standard geothermal application without heat injection and decreasing significantly the reservoir’s temperature.

As different locations for doublets have been chosen, the P-I 1 doublet location is the most promising, where 30 K and 40 K heat injection was used, with a thermal recovery ratio of 0.47 and 0.34, respectively. The 30 K and 40 K of temperature differential allows to increase the operating temperature in the installation and does not cool the reservoir over time. At the same time, these values might be available from waste heat sources.

In terms of thermal recovery ratio, balanced consumption is the best in the 20 K scenario for injection and 20 K for production. In this case, in the doublet P-I 4 receives up to 94% of the injected energy, but achieved temperatures are low. From a practical point of view, the heat storage in a scenario of 30 K or 40 K temperature difference where the temperature of the reservoir increases is preferred. A disadvantageous case is the 10 K scenario, where an extreme cooling of the reservoir occurs, even greater than in the geothermal scenario, where only heat production without injection occurs.

Changing of the reservoir temperature during several phases and particularly increasing temperature may induce some minerals precipitation. Some increasing precipitation potential is shown by aragonite and calcite. This must be considered while planning the infrastructure of the ATES system in the area.

This study shows a potential for the sustainable use of ATES and increase in the geothermal potential in the southern part of the Mogilno–Łódź Trough and may open some opportunities for further ATES developments in the trough. Future research for the optimization of aquifer thermal energy storage and recovery in the area and detailed reservoir parameter examination should be done prior to any investment.

## Supplementary Information

Below is the link to the electronic supplementary material.

Supplementary file1 (DOCX 3226 KB)
