# Supplementary material for: Simulation study of the Lower Cretaceous geothermal reservoir for aquifer thermal energy storage
Source: Environ Geochem Health. 2021 Oct 28;44(7):2253–79. doi: 10.1007/s10653-021-01130-7 (PMC9177501; doi:10.1007/s10653-021-01130-7)
Supplement: Supplementary file 1 — Supplementary file1 (DOCX 3226 KB) [file 10653_2021_1130_MOESM1_ESM.docx]

**Supplementary materials to the article**

**“SIMULATION STUDY OF THE LOWER CRETACEOUS GEOTHERMAL RESERVOIR FOR AQUIFER THERMAL ENERGY STORAGE”**

**by**

**Hałaj E., Pająk L., Papiernik B.**


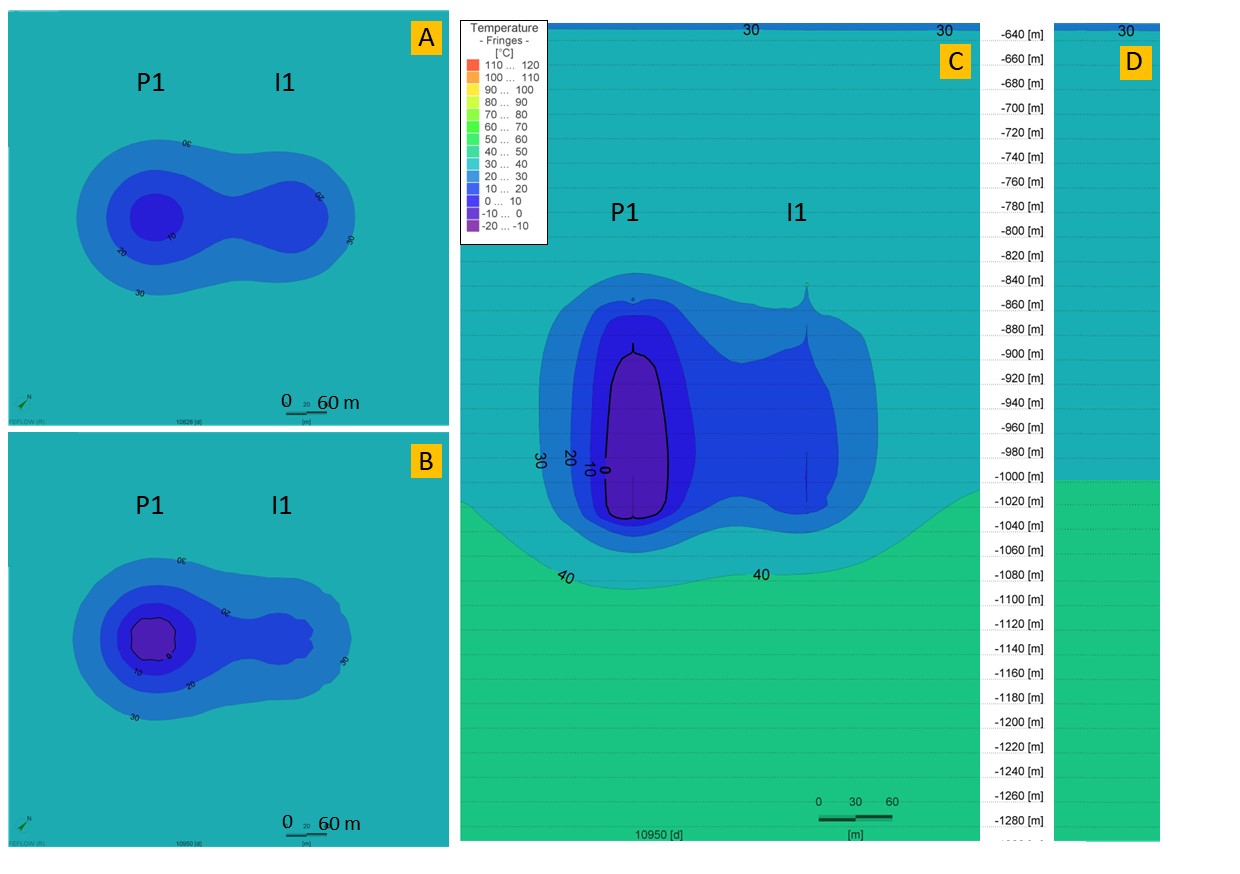


**Fig. A1** Thermal performance of the P-I 1 doublet for 10 K temperature differential on the end of the last, 30^th^ storage phase (A) and at the end of the last year, after the 30^th^ extraction phase (B) and the cross-section view at the end of the 30^th^ extraction phase (C). (D) is the initial temperature profile before wells operation (0 day). The legend is valid for all insets. P 1 and I 1 wells’ plain views located at depths 928.2 m and 917.5 m respectively


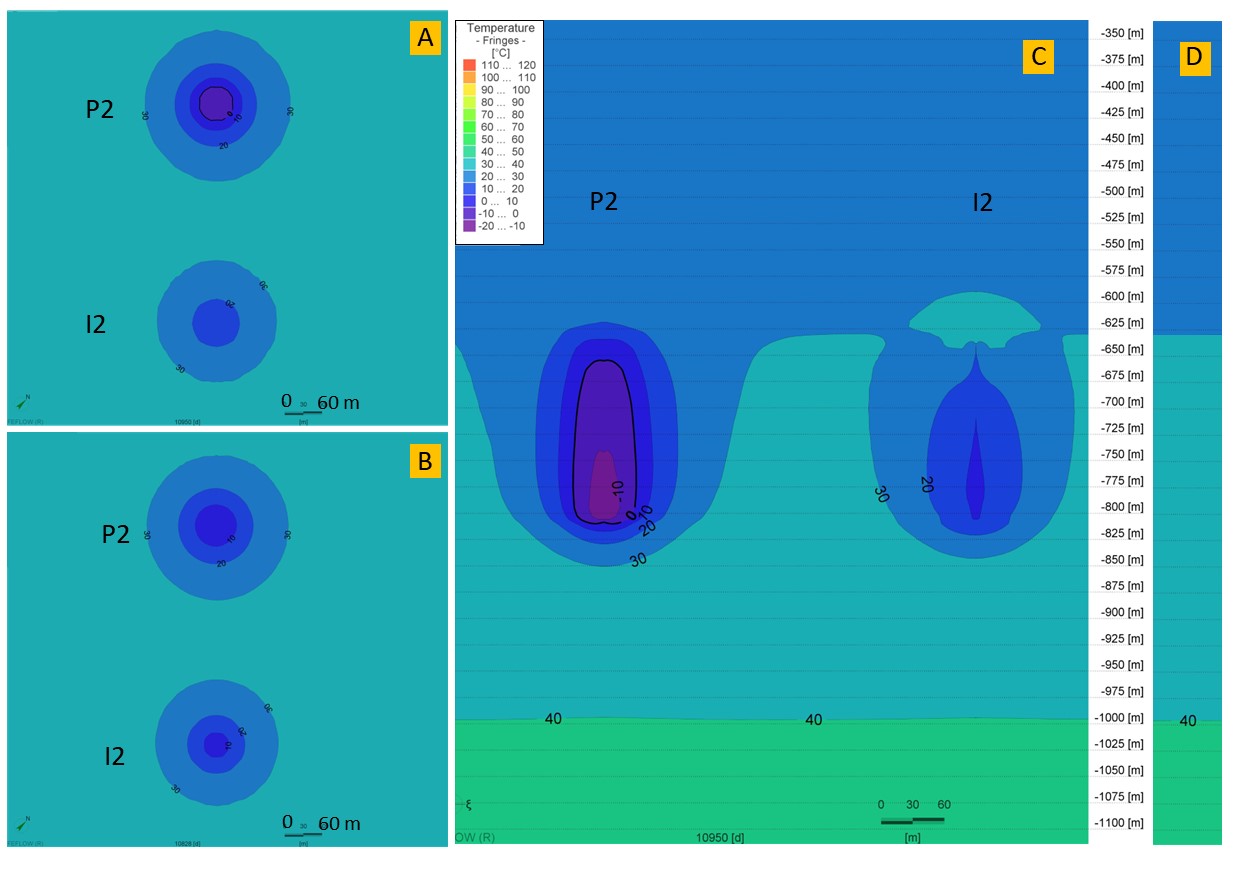


**Fig. A2** Thermal performance of the P-I 2 doublet for 10 K temperature differential on the end of the last, 30^th^ storage phase (A) and at the end of the last year, after the 30^th^ extraction phase (B) and the cross-section view at the end of the 30^th^ extraction phase (C). (D) is the initial temperature profile before wells operation (0 day). The legend is valid for all insets. P 2 and I 2 wells’ plain views located at depths 704.9 m and 711.9 m respectively


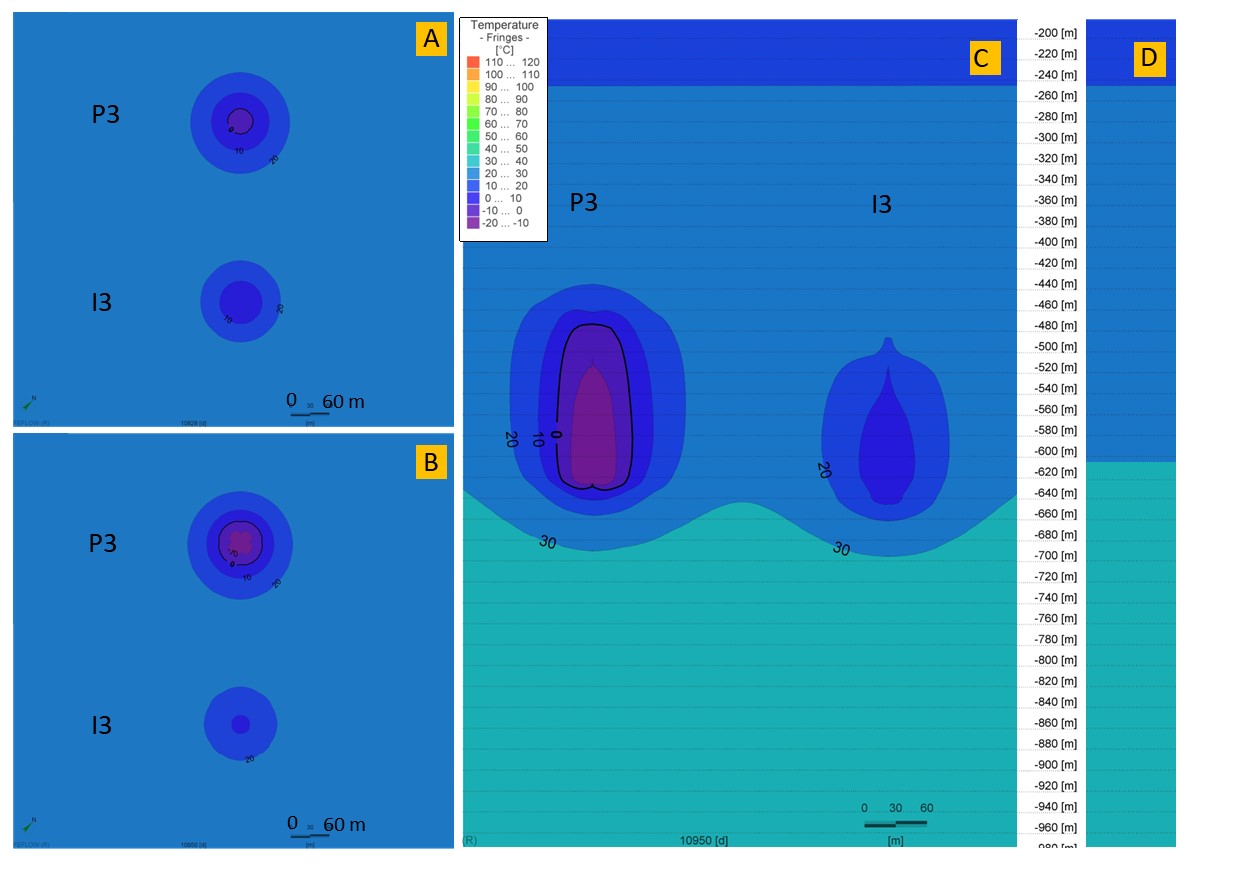


**Fig. A3** The thermal performance of the P-I 3 doublet for 10 K temperature differential on the end of the last, 30^th^ storage phase (A) and at the end of the last year, after the 30^th^ extraction phase (B) and the cross-section view at the end of the 30^th^ extraction phase (C). (D) is the initial temperature profile before wells operation (0 day). The legend is valid for all insets. P 3 and I 3 wells’ plain views located at depths 532.1 m and 552.9 m respectively


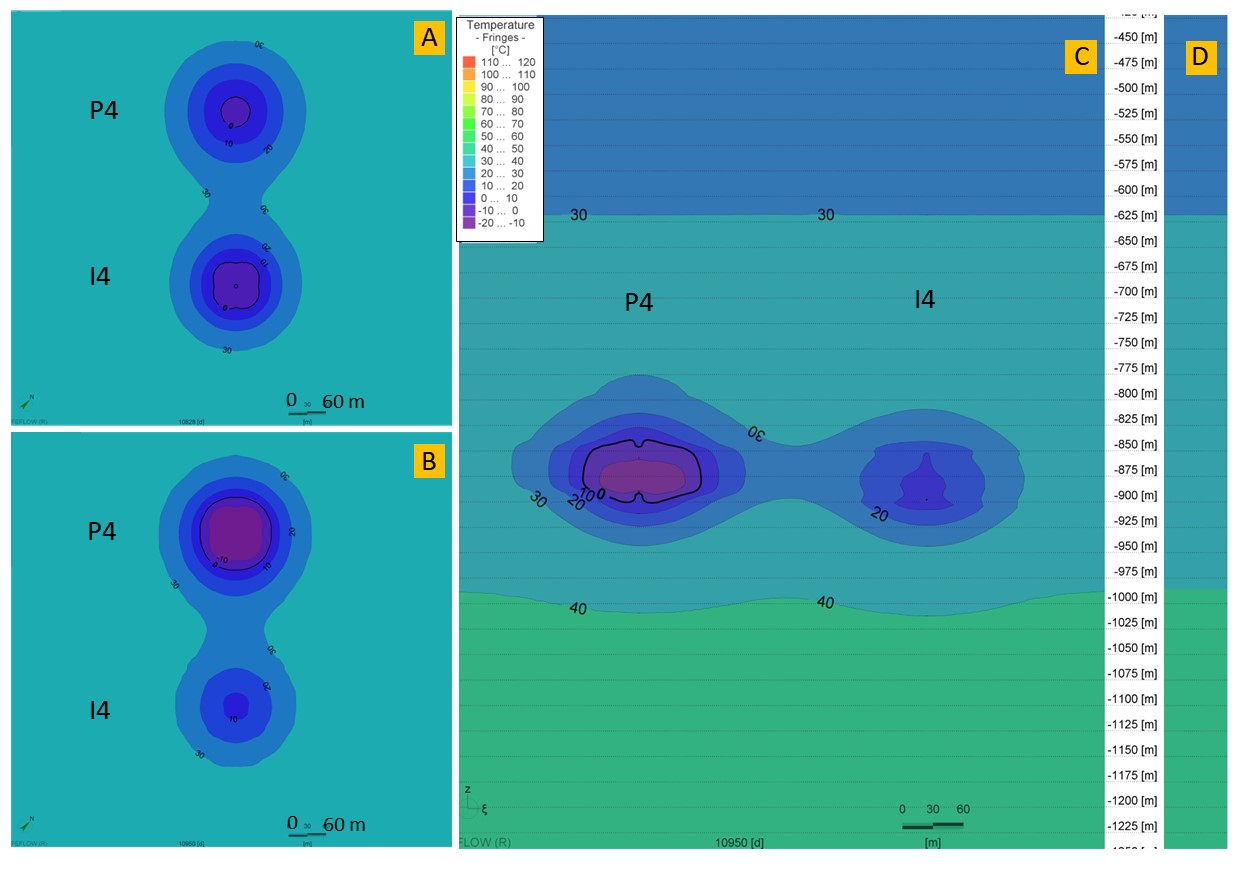


**Fig. A4** Thermal performance of the P-I 4 doublet for 10 K temperature differential on the end of the last, 30^th^ storage phase (A) and at the end of the last year, after the 30^th^ extraction phase (B) and the cross-section view at the end of the 30^th^ extraction phase (C). (D) is the initial temperature profile before wells operation (0 day). The legend is valid for all insets. P 4 and I 4 wells’ plain views located at depths 878.0 m and 886.9 m respectively


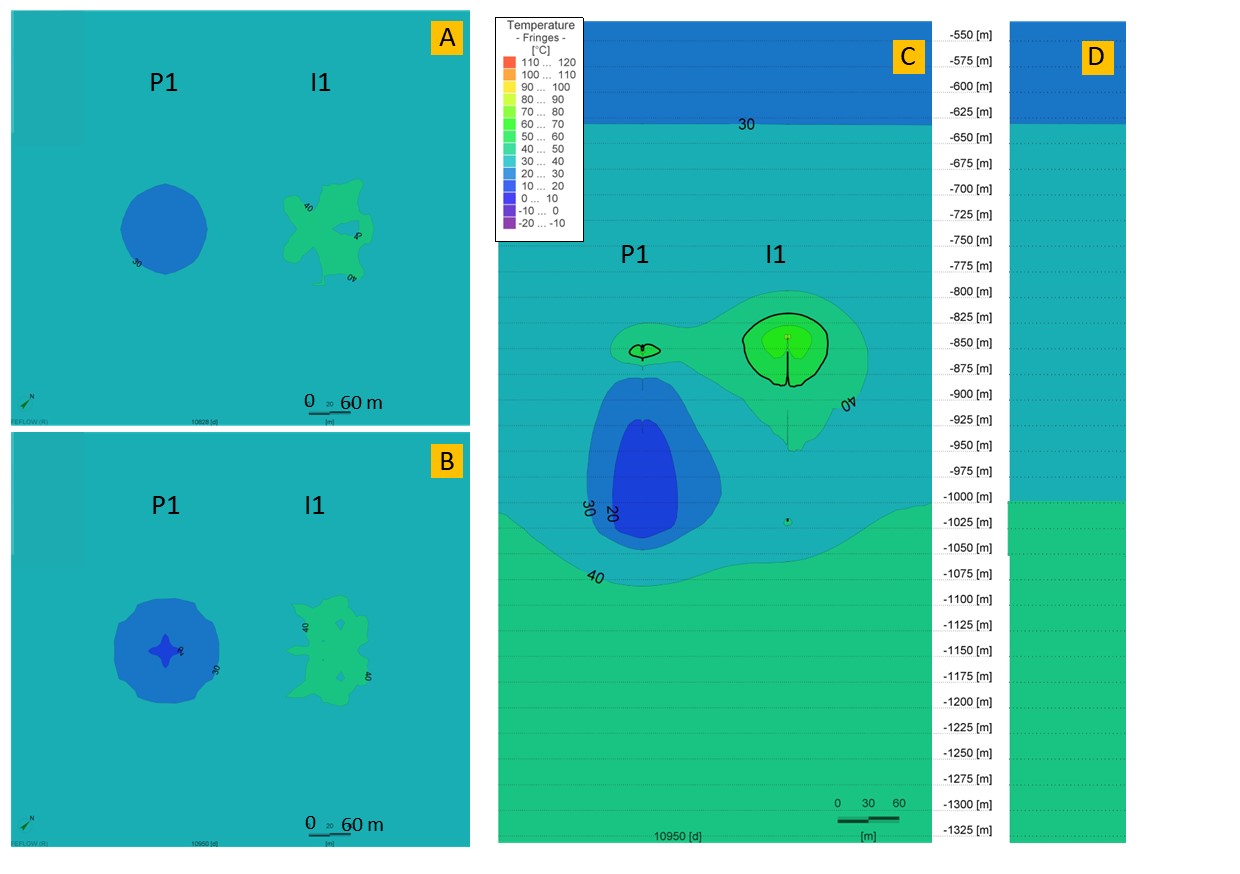


**Fig. B1** Thermal performance of the P-I 1 doublet for 20 K temperature differential on the end of the last, 30^th^ storage phase (A) and at the end of the last year, after the 30^th^ extraction phase (B) and the cross-section view at the end of the 30^th^ extraction phase (C). (D) is the initial temperature profile before wells operation (0 day). The legend is valid for all insets. P 1 and I 1 wells’ plain views located at depths 928.2 m and 917.5 m respectively


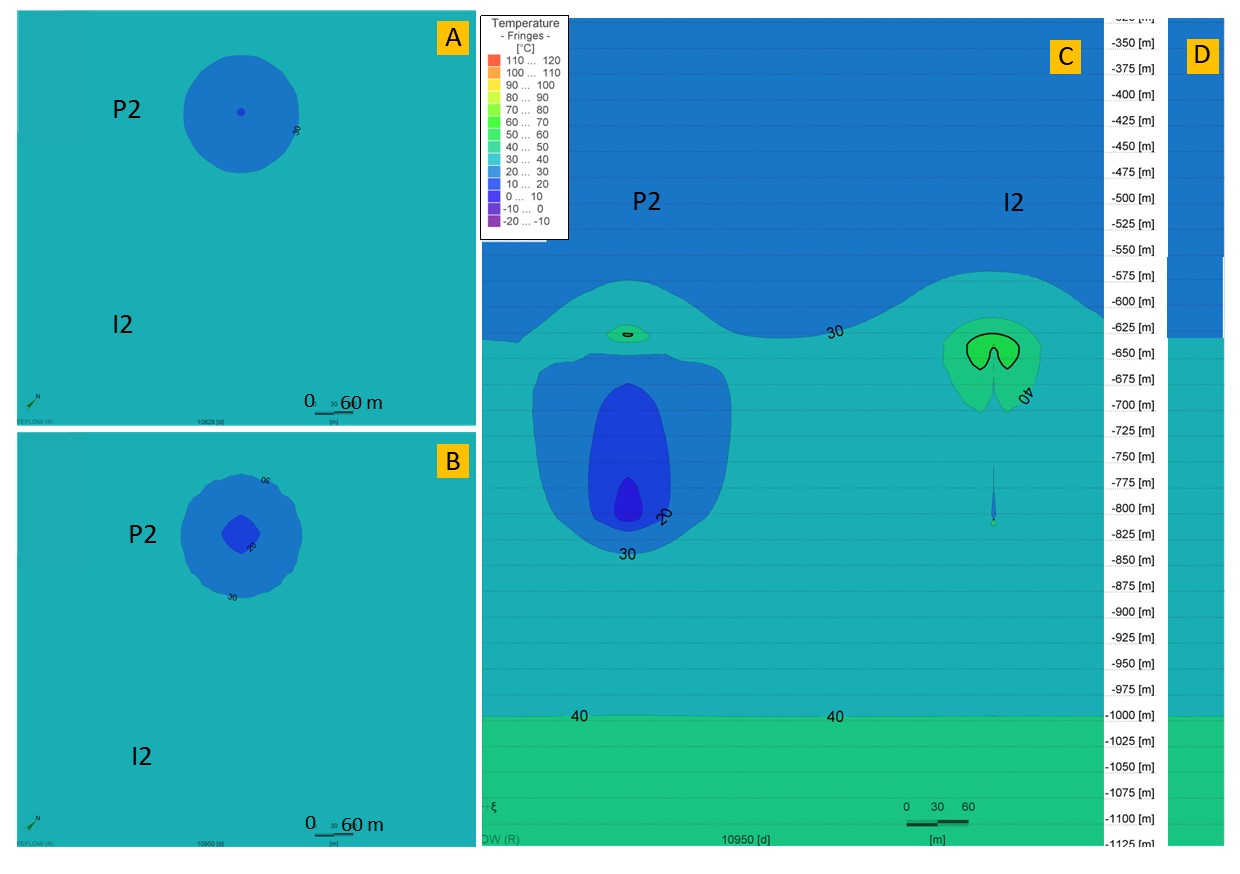


**Fig. B2** Thermal performance of the P-I 2 doublet for 20 K temperature differential on the end of the last, 30^th^ storage phase (A) and at the end of the last year, after the 30^th^ extraction phase (B) and the cross-section view at the end of the 30^th^ extraction phase (C). (D) is the initial temperature profile before wells operation (0 day). The legend is valid for all insets. P 2 and I 2 wells’ plain views located at depths 704.9 m and 711.9 m respectively


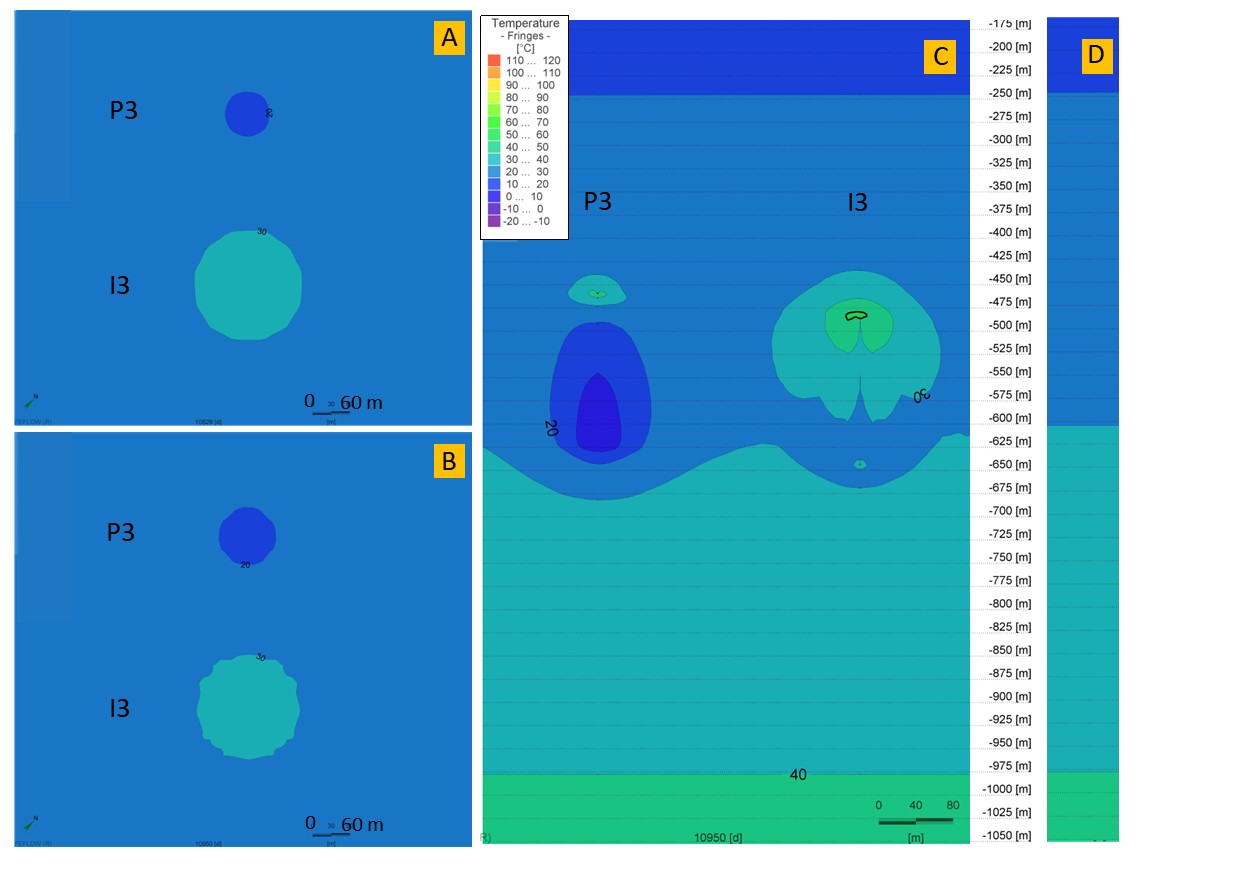


**Fig. B3** Thermal performance of the P-I 3 doublet for 20 K temperature differential on the end of the last, 30^th^ storage phase (A) and at the end of the last year, after the 30^th^ extraction phase (B) and the cross-section view at the end of the 30^th^ extraction phase (C). (D) is the initial temperature profile before wells operation (0 day). The legend is valid for all insets. P 3 and I 3 wells’ plain views located at depths 532.1 m and 552.9 m respectively


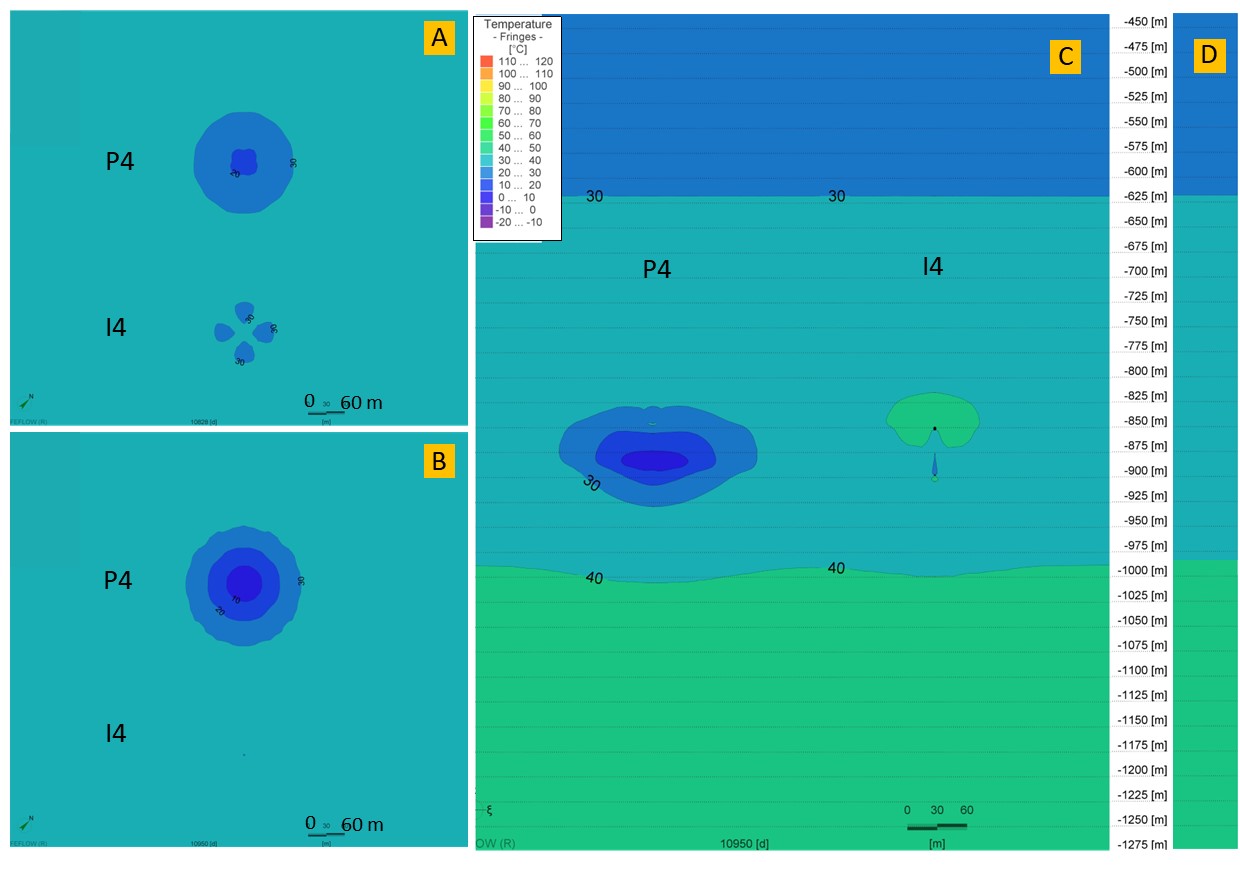


**Fig. B4** Thermal performance of the P-I 4 doublet for 20 K temperature differential on the end of the last, 30^th^ storage phase (A) and at the end of the last year, after the 30^th^ extraction phase (B) and the cross-section view at the end of the 30^th^ extraction phase (C). (D) is the initial temperature profile before wells operation (0 day). The legend is valid for all insets. P 4 and I 4 wells’ plain views located at depths 878.0 m and 886.9 m respectively


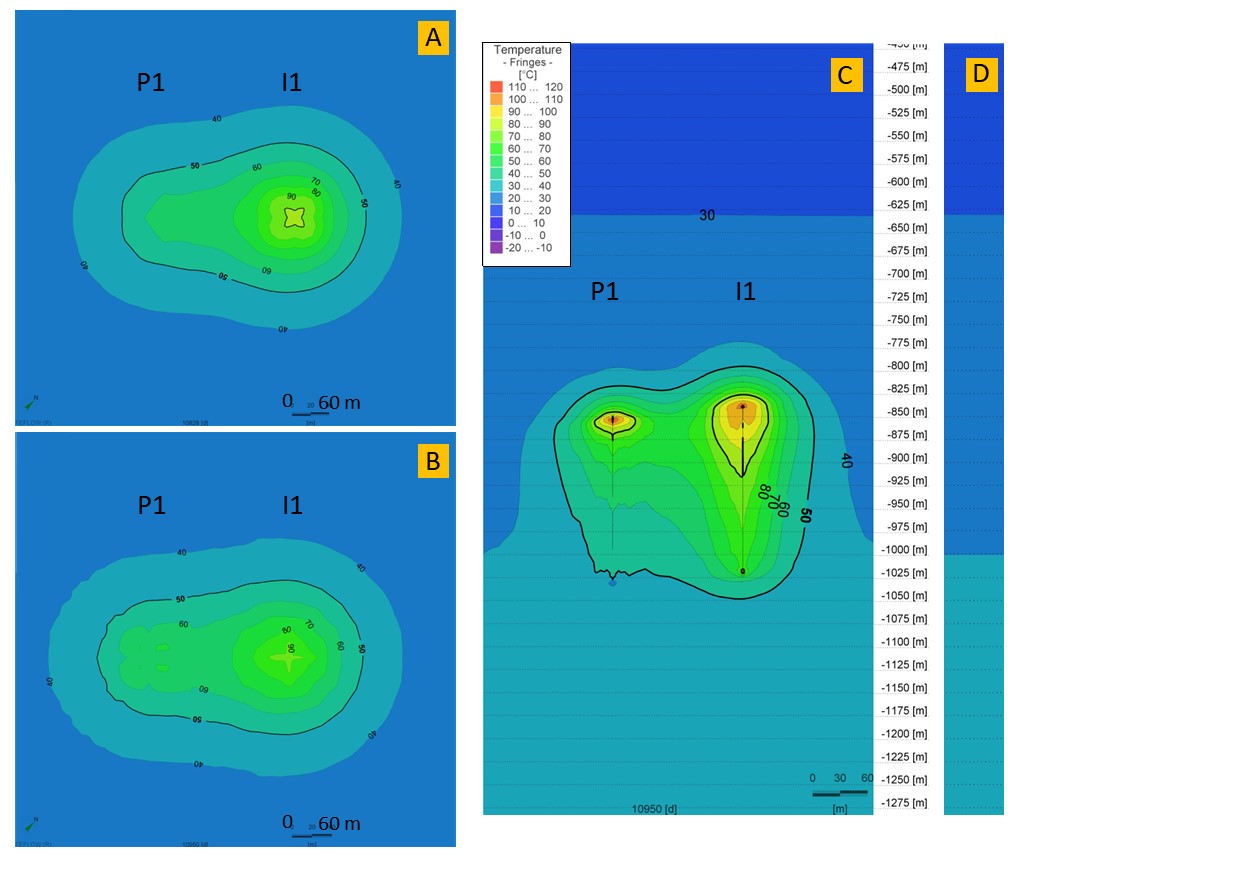


**Fig. C1** Thermal performance of the P-I 1 doublet for 40 K temperature differential on the end of the last, 30^th^ storage phase (A) and at the end of the last year, after the 30^th^ extraction phase (B) and the cross-section view at the end of the 30^th^ extraction phase (C). (D) is the initial temperature profile before wells operation (0 day). The legend is valid for all insets. P 1 and I 1 wells’ plain views located at depths 928.2 m and 917.5 m respectively


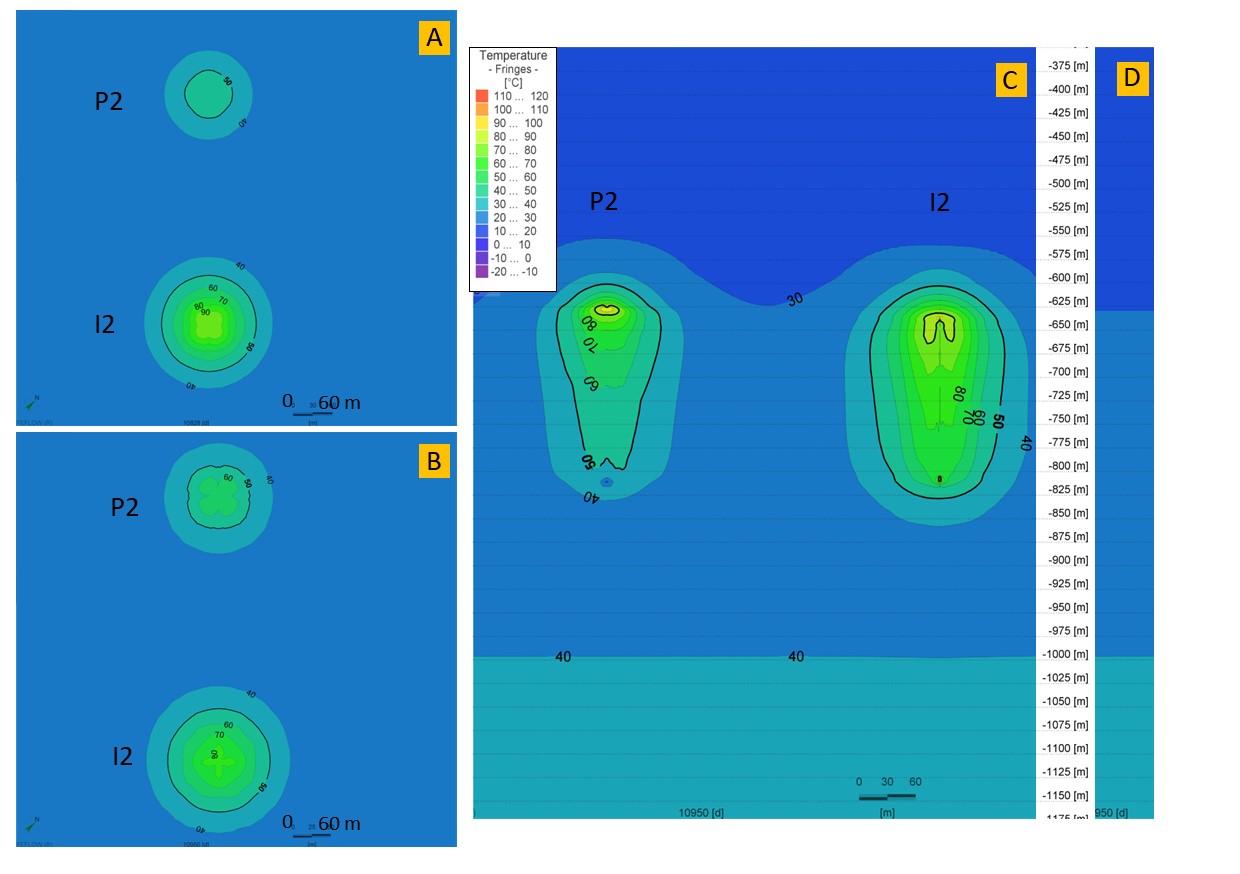


**Fig. C2** Thermal performance of the P-I 2 doublet for 40 K temperature differential on the end of the last, 30^th^ storage phase (A) and at the end of the last year, after the 30^th^ extraction phase (B) and the cross-section view at the end of the 30^th^ extraction phase (C). (D) is the initial temperature profile before wells operation (0 day). The legend is valid for all insets. P 2 and I 2 wells’ plain views located at depths 704.9 m and 711.9 m respectively


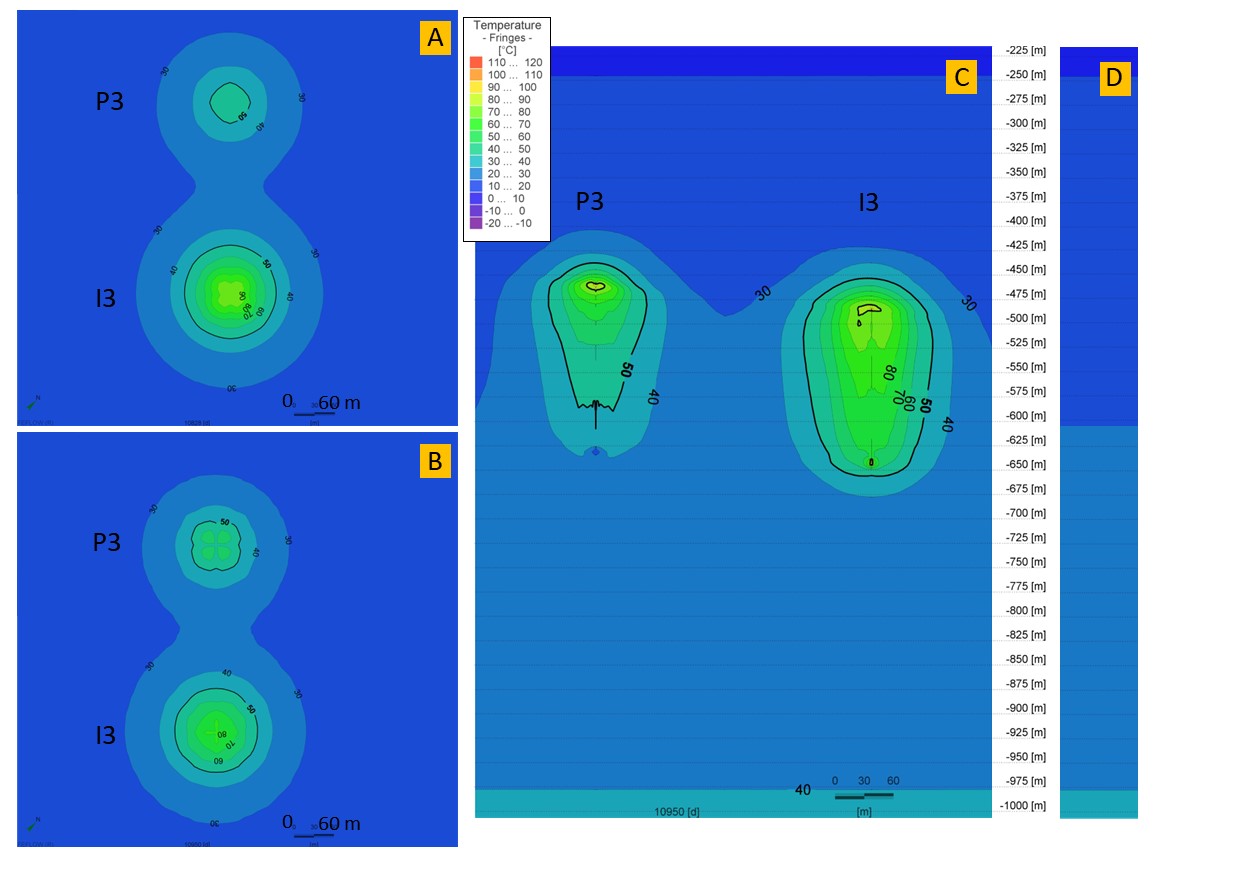


**Fig. C3** The thermal performance of the P-I 3 doublet for 40 K temperature differential on the end of the last, 30^th^ storage phase (A) and at the end of the last year, after the 30^th^ extraction phase (B) and the cross-section view at the end of the 30^th^ extraction phase (C). (D) is the initial temperature profile before wells operation (0 day). The legend is valid for all insets. P 3 and I 3 wells’ plain views located at depths 532.1 m and 552.9 m respectively


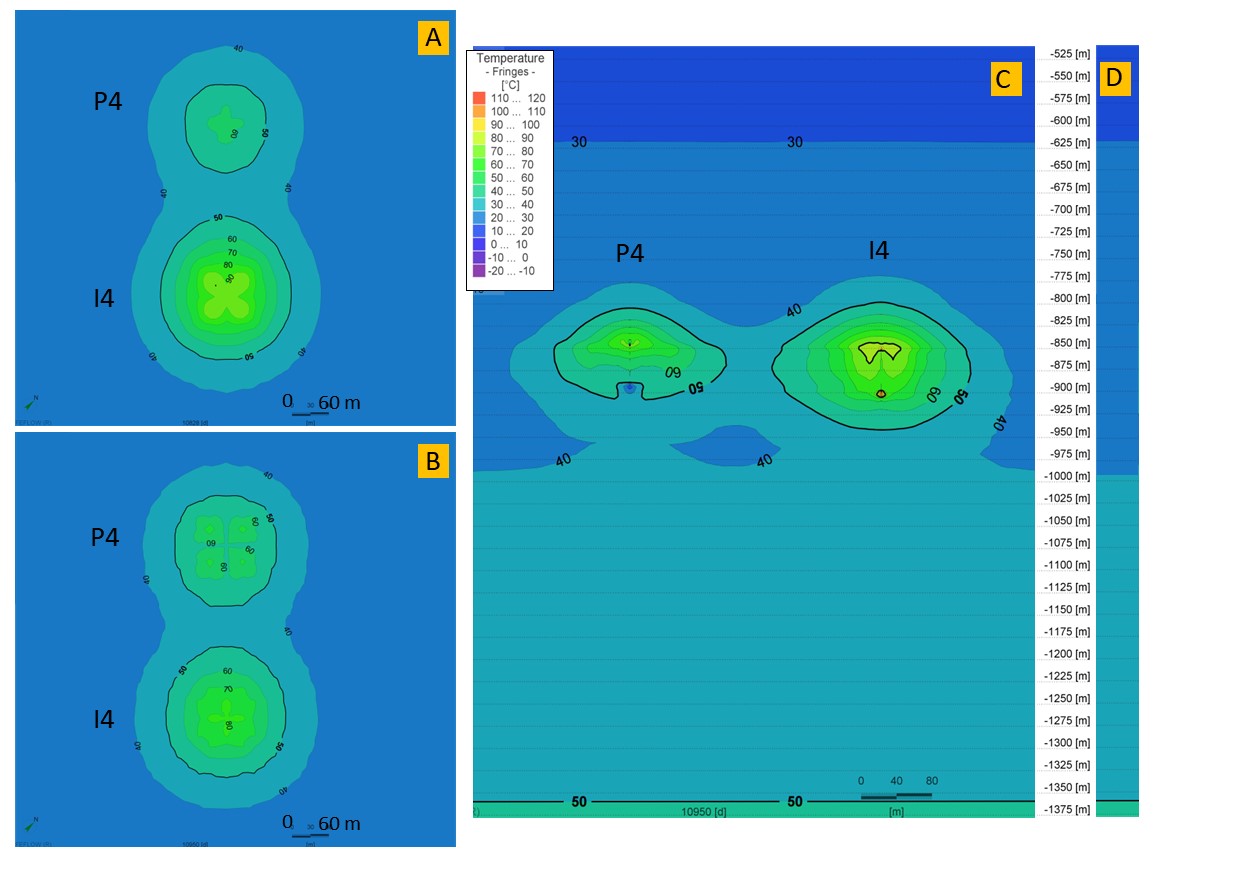


**Fig. C4** Thermal performance of the P-I 4 doublet for 40 K temperature differential on the end of the last, 30^th^ storage phase (A) and at the end of the last year, after the 30^th^ extraction phase (B) and the cross-section view at the end of the 30^th^ extraction phase (C). (D) is the initial temperature profile before wells operation (0 day). The legend is valid for all insets. P 4 and I 4 wells’ plain views located at depths 878.0 m and 886.9 m respectively


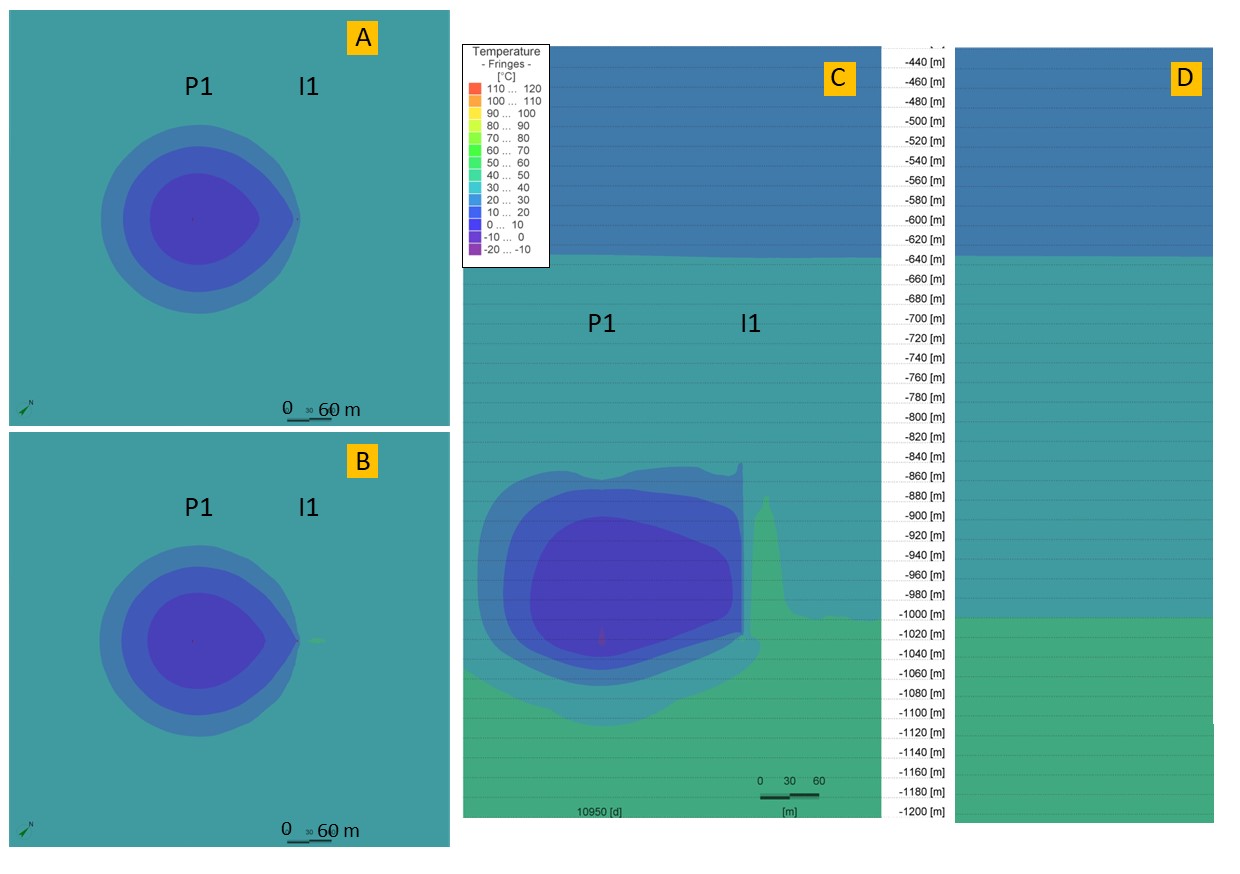


**Fig. D1** Thermal performance of the P-I 1 doublet for geothermal heating scenario on the end of the last, 30^th^ storage phase (A) and at the end of the last year, after the 30^th^ extraction phase (B) and the cross-section view at the end of the 30^th^ extraction phase (C). (D) is the initial temperature profile before wells operation (0 day). The legend is valid for all insets. P 1 and I 1 wells’ plain views located at depths 928.2 m and 917.5 m respectively


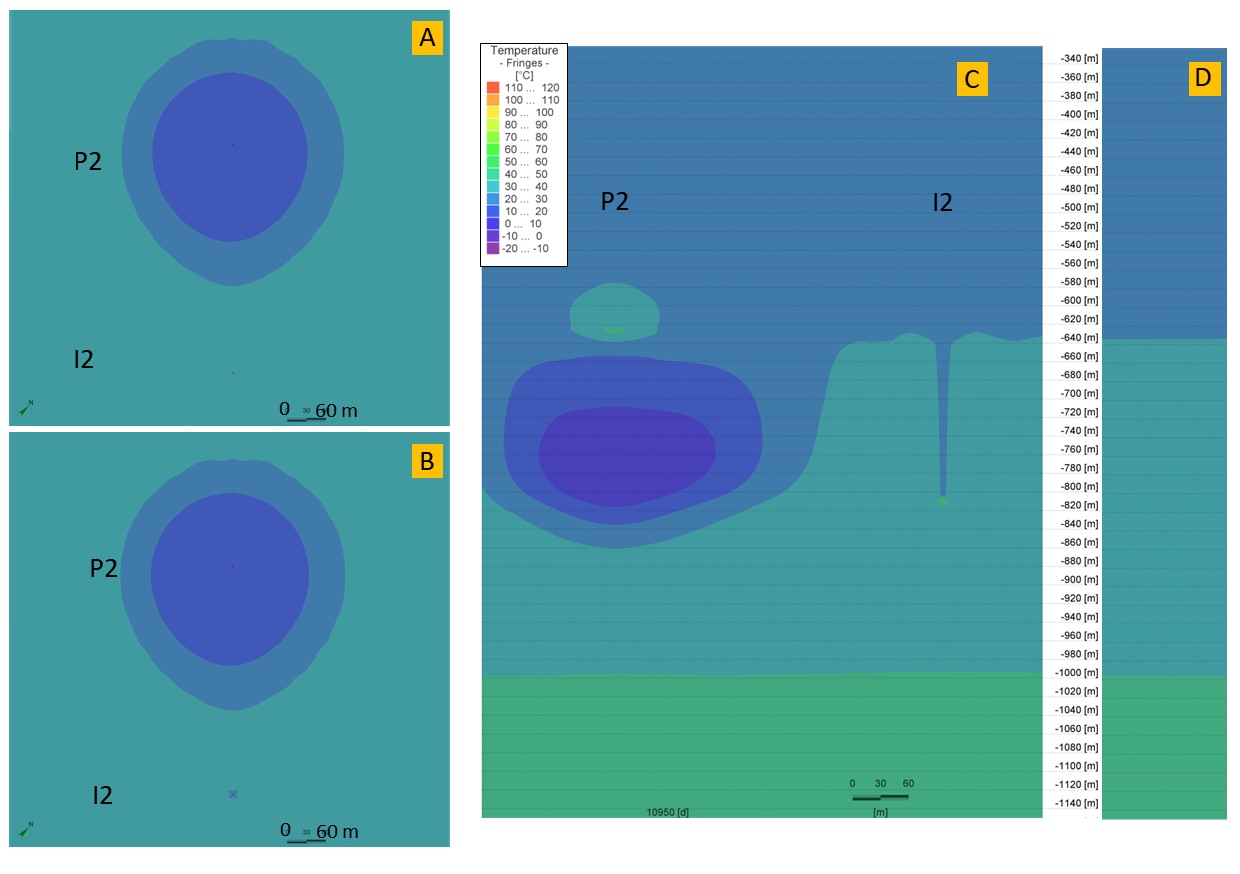


**Fig. D2** Thermal performance of the P-I 2 doublet for geothermal heating scenario on the end of the last, 30^th^ storage phase (A) and at the end of the last year, after the 30^th^ extraction phase (B) and the cross-section view at the end of the 30^th^ extraction phase (C). (D) is the initial temperature profile before wells operation (0 day). The legend is valid for all insets. P 2 and I 2 wells’ plain views located at depths 704.9 m and 711.9 m respectively


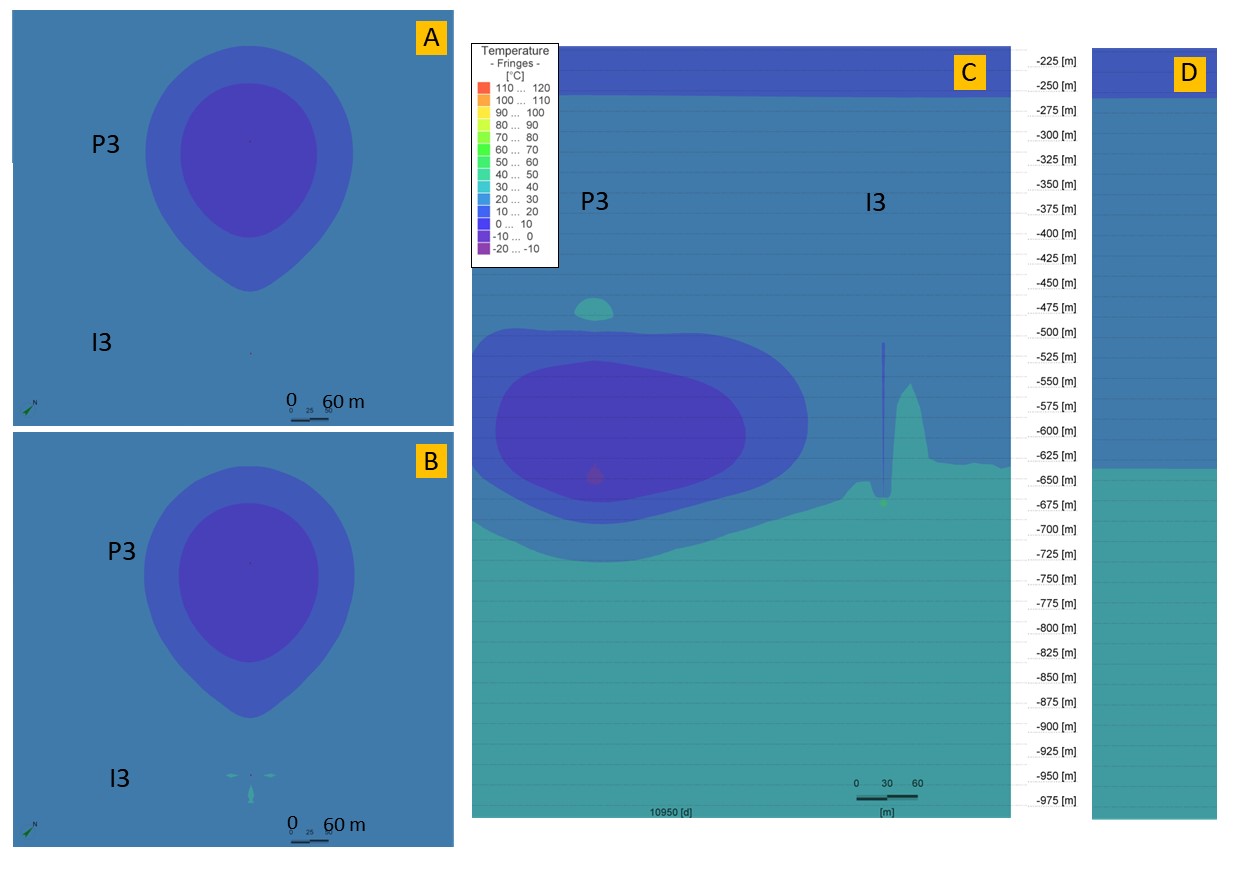


**Fig. D3** Thermal performance of the P-I 3 doublet for geothermal heating scenario on the end of the last, 30^th^ storage phase (A) and at the end of the last year, after the 30^th^ extraction phase (B) and the cross-section view at the end of the 30^th^ extraction phase (C). (D) is the initial temperature profile before wells operation (0 day). The legend is valid for all insets. P 3 and I 3 wells’ plain views located at depths 532.1 m and 552.9 m respectively.


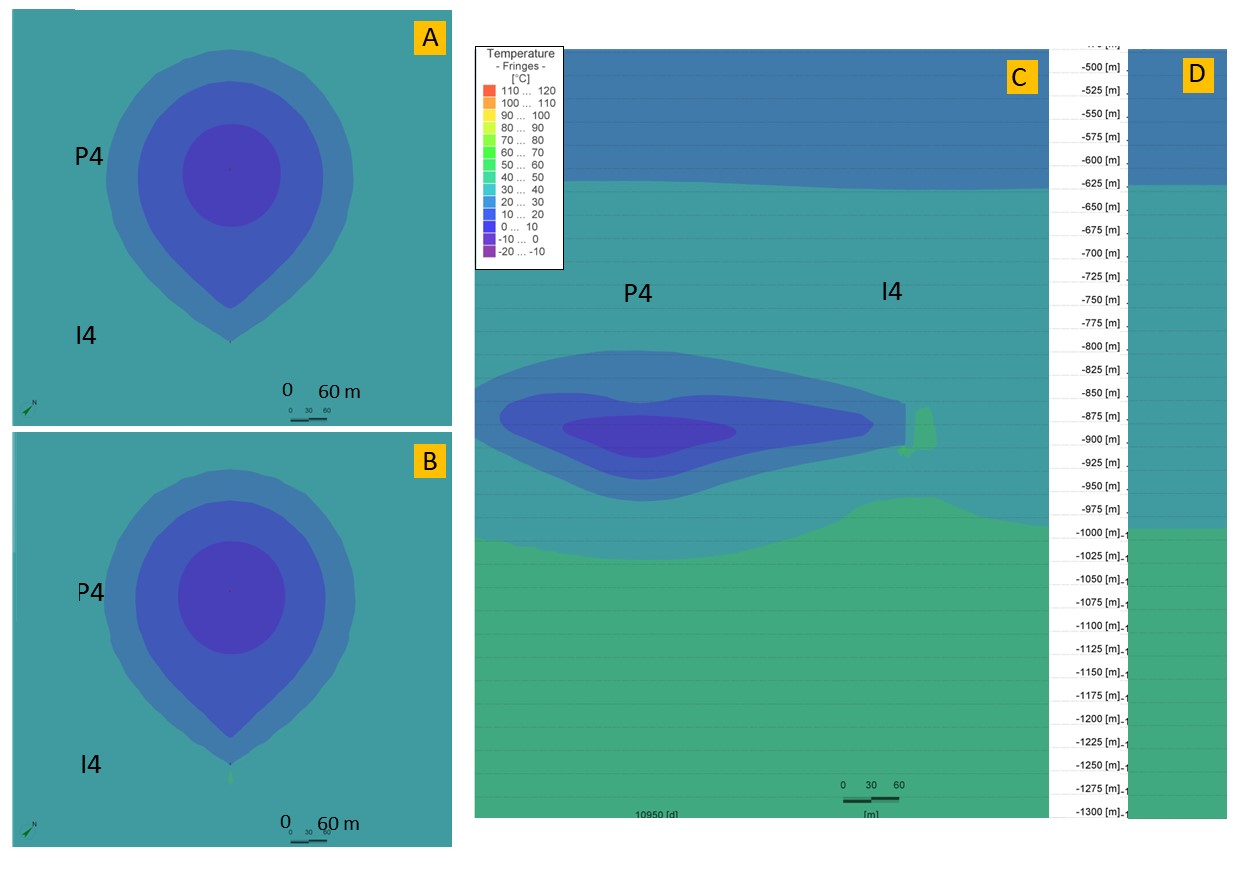


**Fig. D4** Thermal performance of the P-I 4 doublet for geothermal heating scenario on the end of the last, 30^th^ storage phase (A) and at the end of the last year, after the 30^th^ extraction phase (B) and the cross-section view at the end of the 30^th^ extraction phase (C). (D) is the initial temperature profile before wells operation (0 day). The legend is valid for all insets. P 4 and I 4 wells’ plain views located at depths 878.0 m and 886.9 m respectively


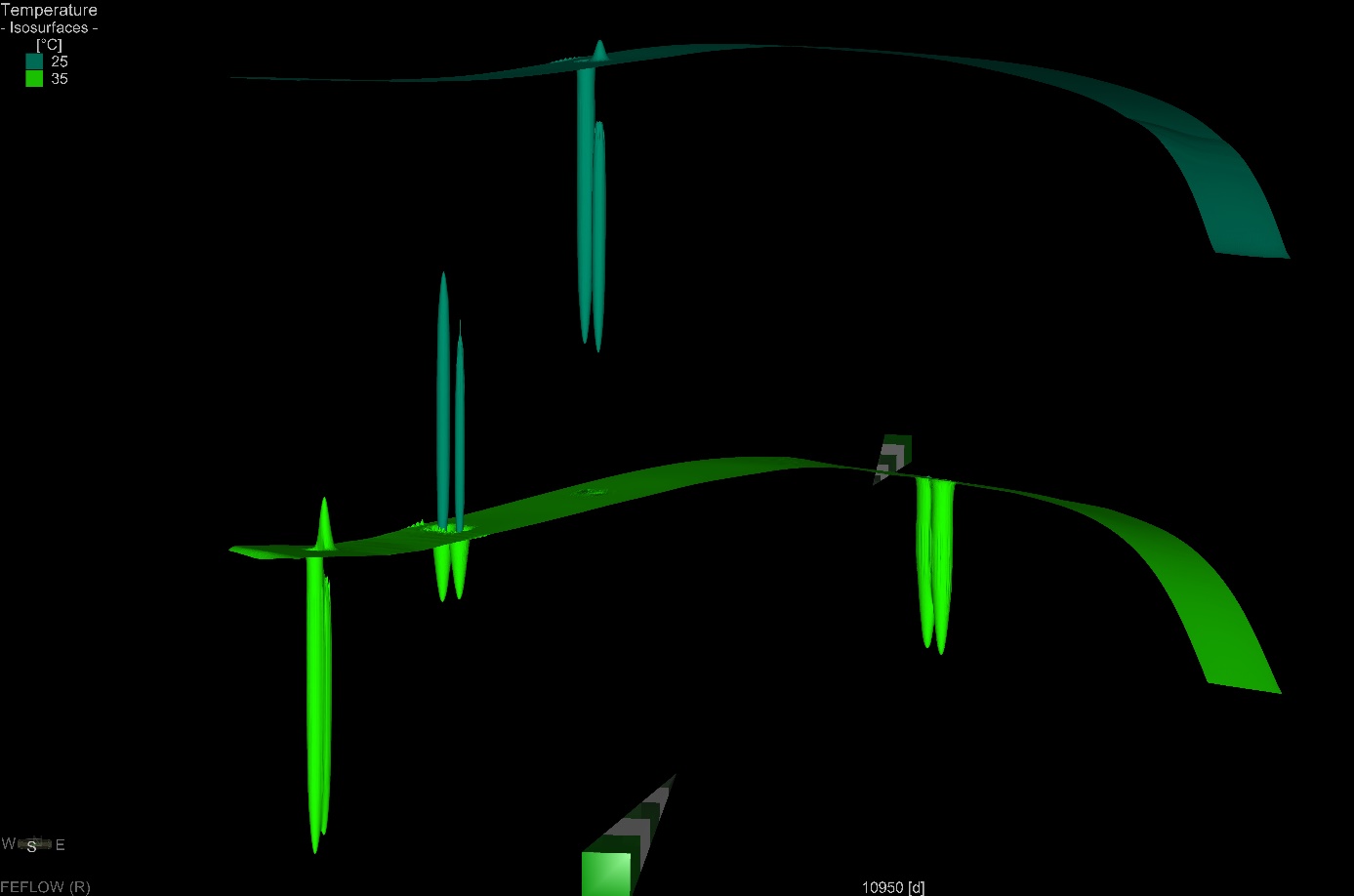


**Fig. E1** 3D visualization of 25 and 35 °C isosurfaces for the ATES simulation within 10 K temperature differential scenario after 30 years


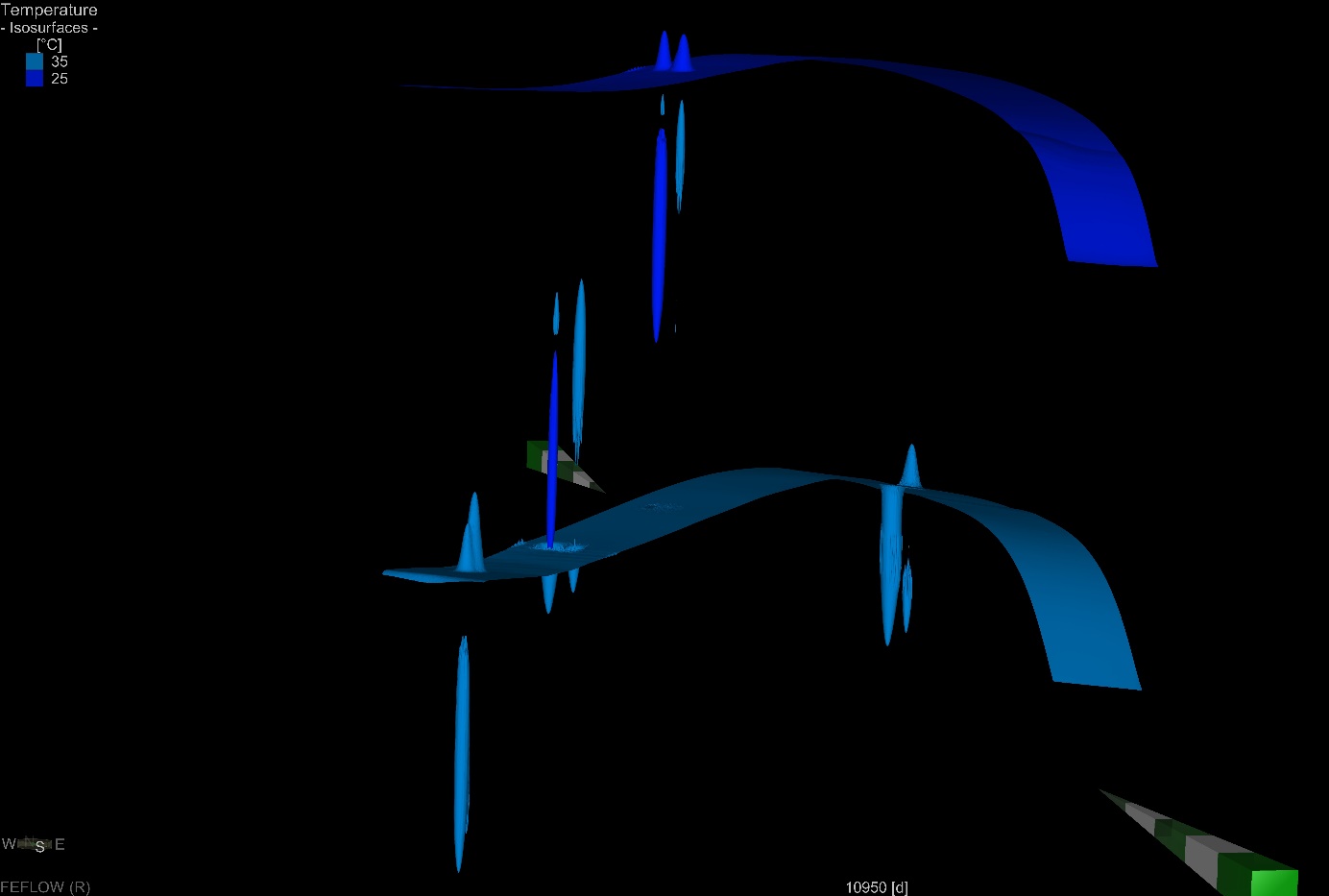


**Fig. E2** 3D visualization of 25 and 35 °C isosurfaces for the ATES simulation within 20 K temperature differential scenario after 30 years


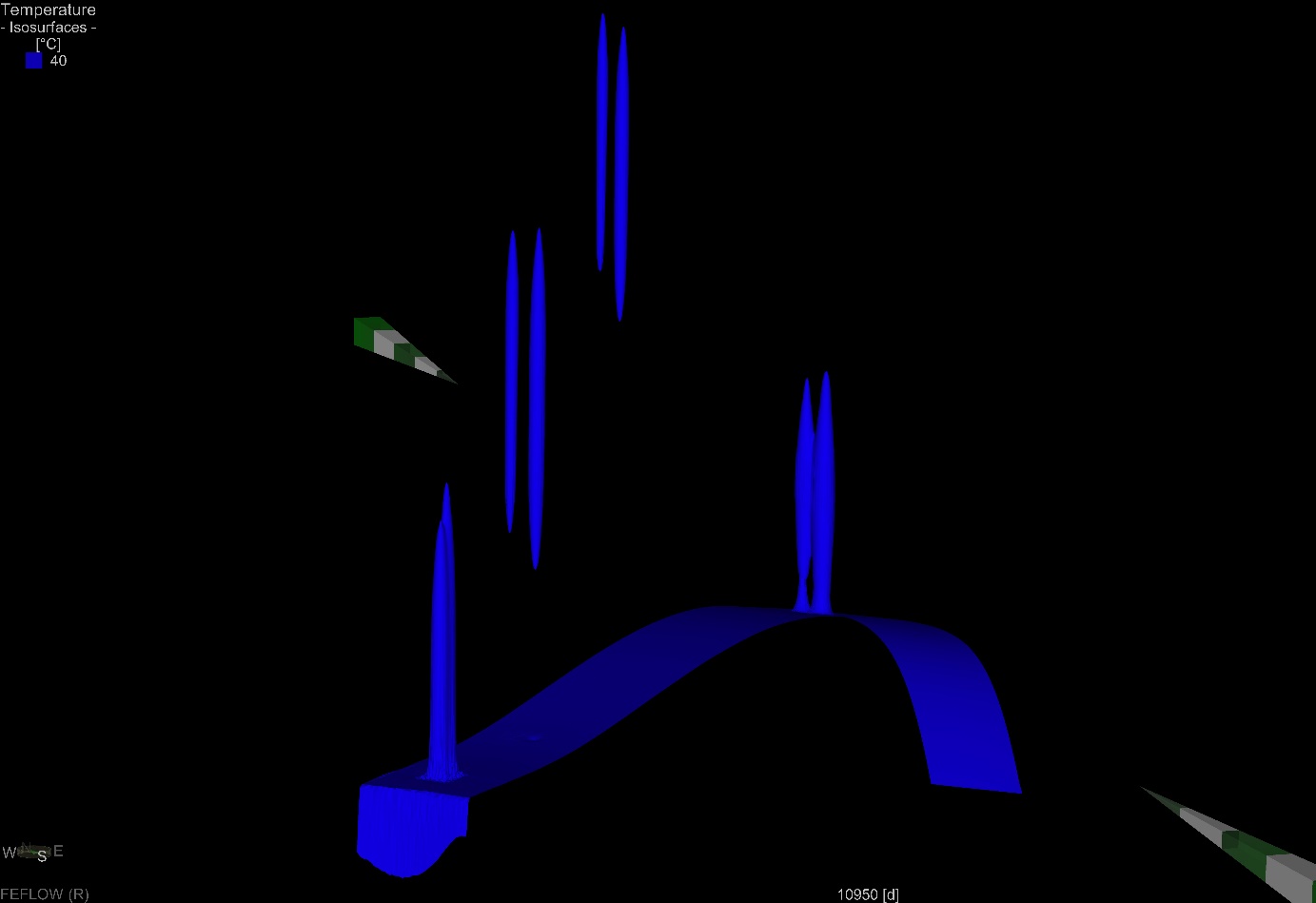


**Fig. E3** 3D visualization of 40 °C isosurface for the ATES simulation within 40 K temperature differential scenario after 30 years


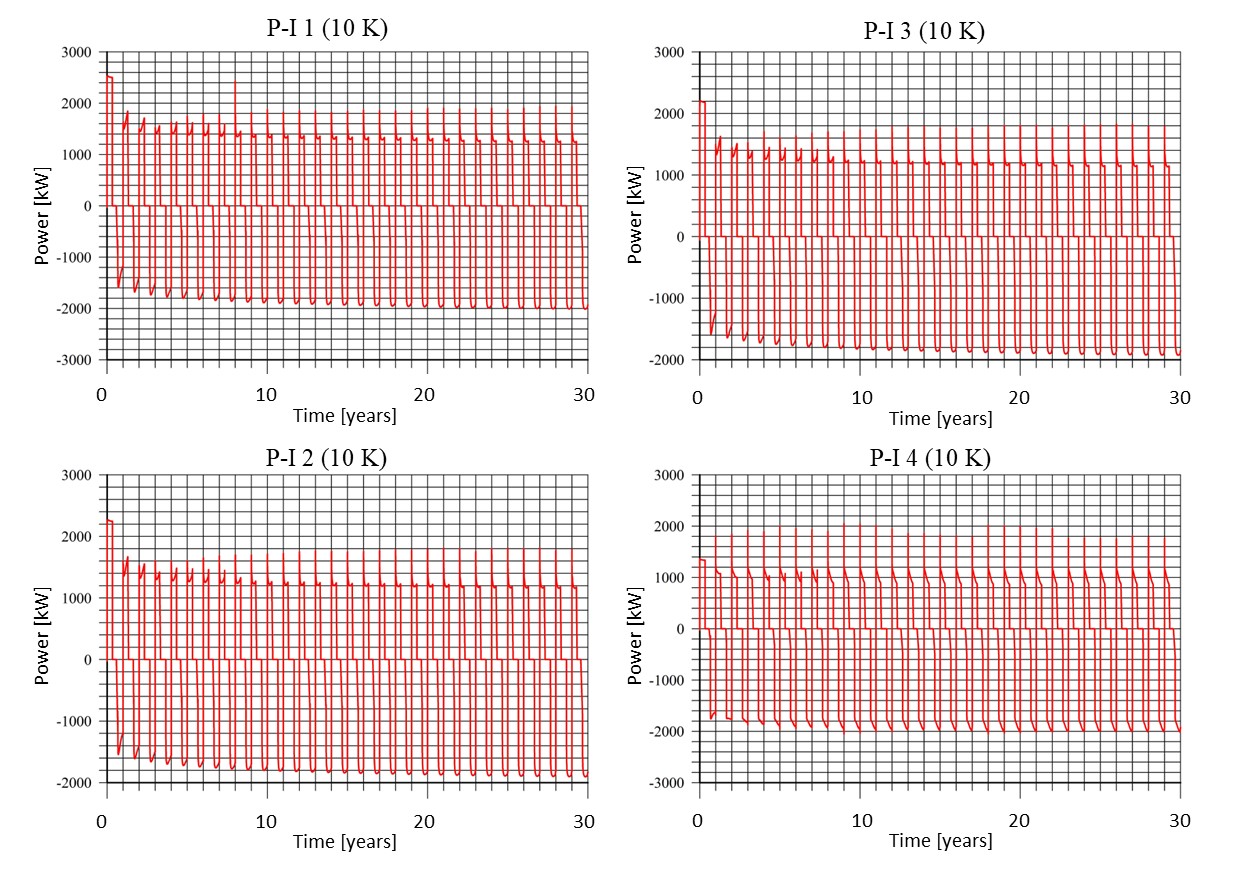


**Fig. F1** Thermal power for the P-I 1-4 doublets in the 30 years of operation for the 10 K temperature differential asset. Each year consists of 3 phases: injection until the 121^st^ day, storage until the 243^rd^ day, and extraction until the 365^th^ day


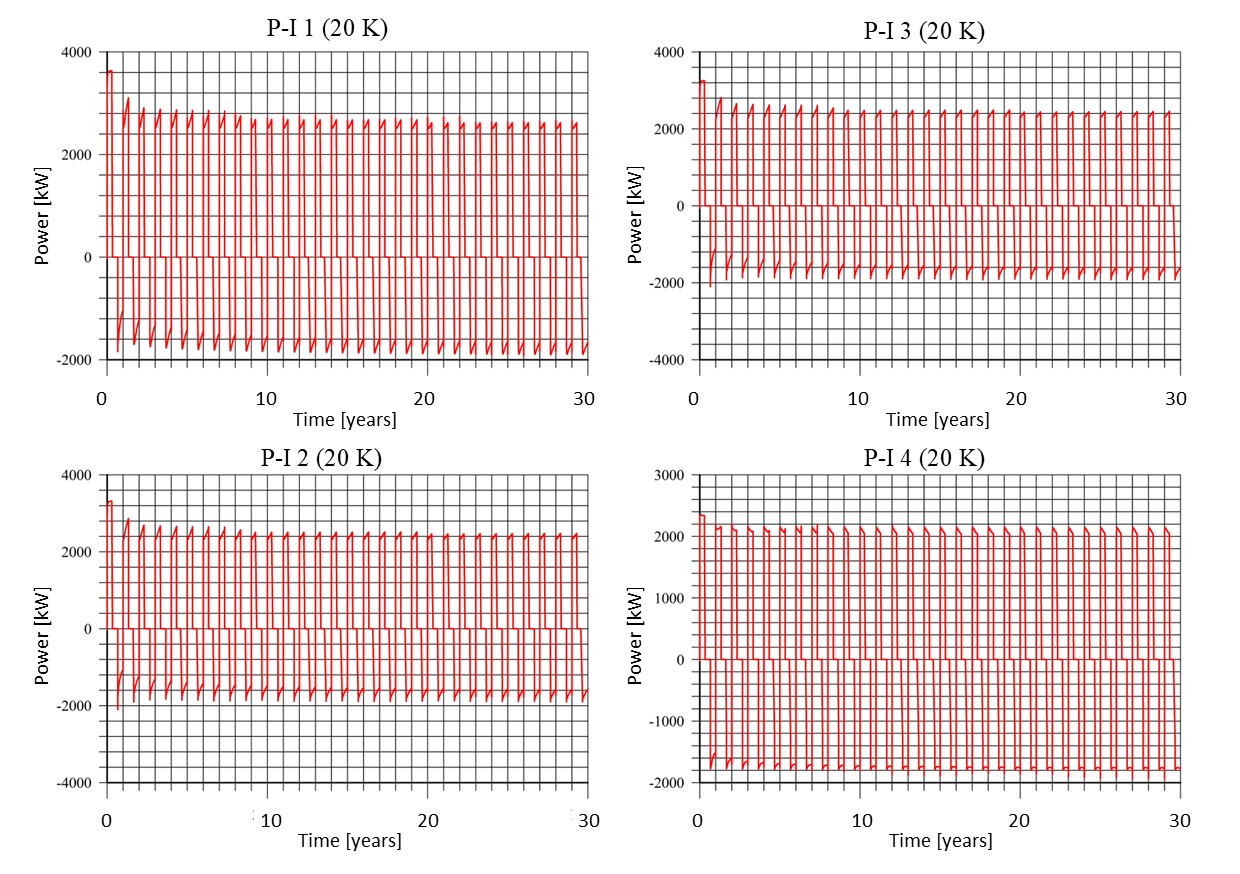


**Fig. F2** Thermal power for the P-I 1-4 doublets in the 30 years of operation for the 20 K temperature differential asset. Each year consists of 3 phases: injection until the 121^st^ day, storage until the 243^rd^ day, and extraction until the 365^th^ day


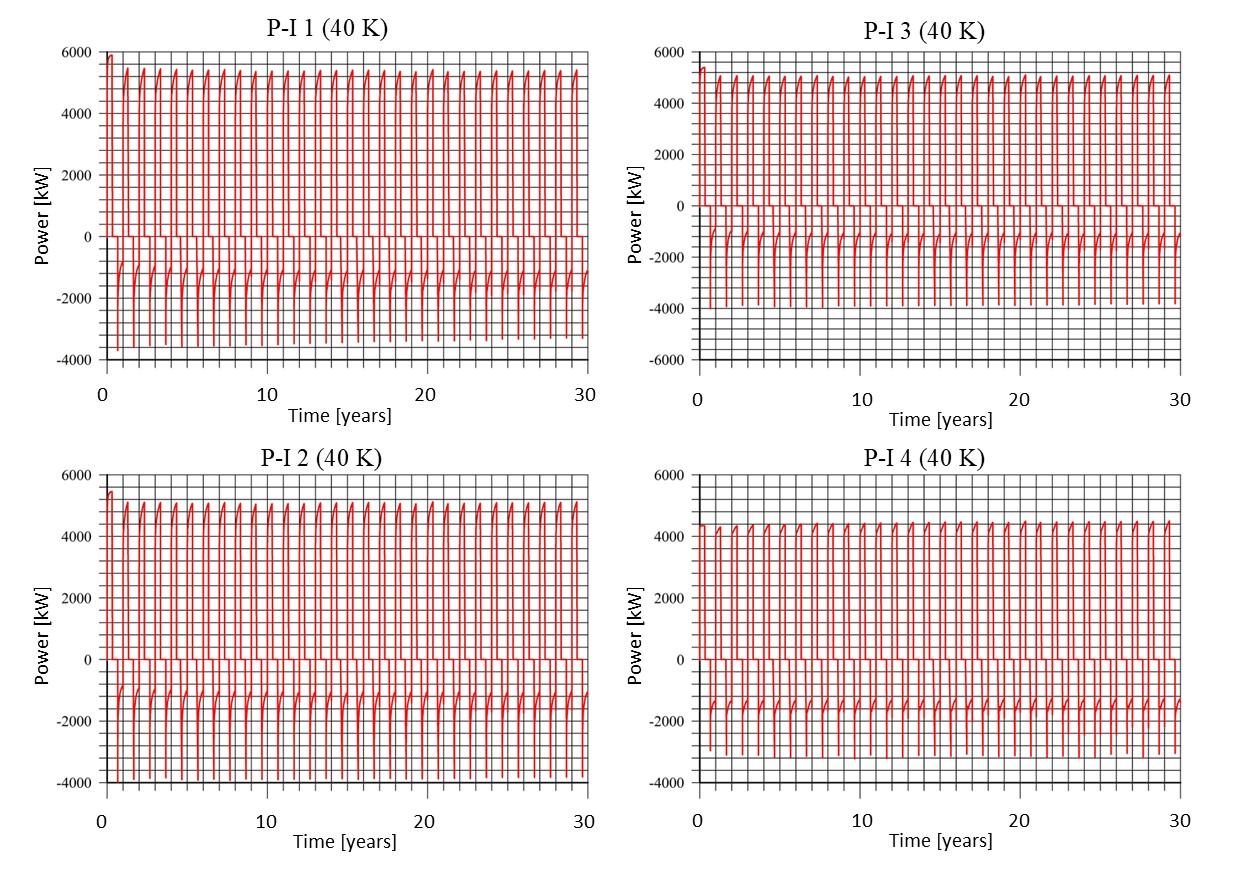


**Fig. F3** Thermal power for the P-I 1-4 doublets in the 30 years of operation for the 40 K temperature differential asset. Each year consists of 3 phases: injection until the 121^st^ day, storage until the 243^rd^ day, and extraction until the 365^th^ day
